# Supplementary material for: Obama chez moi! The invasion of metropolitan France by the land planarian Obama nungara (Platyhelminthes, Geoplanidae)
Source: PeerJ. 2020 Feb 6;8:e8385. doi: 10.7717/peerj.8385 (PMC7007977; doi:10.7717/peerj.8385)
Supplement: Supplemental Information 4 — Obama chez moi ! L’invasion de la France métropolitaine par le ver plat Obama nungara (Plathelminthes, Geoplanidae). [file peerj-08-8385-s004.pdf]

Obama chez moi ! L'invasion de la France métropolitaine par le ver plat *Obama nungara* (Plathelminthes, Geoplanidae)

Jean-Lou Justine [1], Leigh Winsor [2], Delphine Gey [3], Pierre Gros [4], Jessica Thévenot [5]

1 ISYEB, Institut de Systématique Évolution Biodiversité, UMR7205 CNRS, EPHE, MNHN, UPMC, Université des Antilles, Muséum National d'Histoire Naturelle, Paris, France

2 College of Science and Engineering, James Cook University, Townsville, Queensland, Australia

3 Service de Systématique moléculaire, Muséum National d'Histoire Naturelle, Paris, France

4 Naturaliste amateur, Cagnes-sur-Mer, France

5 UMS Patrinat (CNRS - AFB - MNHN), Muséum National d'Histoire Naturelle, Paris, France

Auteur de correspondance : Jean-Lou Justine, justine@mnhn.fr

Ce texte est la traduction intégrale en français de l'article :

***Obama chez moi*! The invasion of metropolitan France by the land planarian *Obama nungara* (Platyhelminthes, Geoplanidae)**

Publié dans PeerJ, 2020, http ://dx.doi.org/10.7717/peerj.8385

Licence : CC-BY 4.0 (comme le texte original)

Résumé

Contexte

Obama nungara est une espèce de ver plat terrestre originaire d'Amérique du Sud ; l'espèce a été récemment décrite et distinguée d'une espèce similaire, *Obama marmorata*. *Obama nungara* a envahi plusieurs pays d'Europe, mais l'étendue de l'invasion n'a pas été complètement cartographiée.

Méthodes

Dans cet article, sur la base d'une enquête de science participative de cinq ans et demi, qui a fourni 530 signalements de 2013 à 2018, nous avons analysé des informations sur l'invasion de la France métropolitaine par *O. nungara*. Nous avons également étudié la variabilité des séquences de cytochrome c oxydase 1 (COI), nouvellement obtenues de spécimens de France, Italie et Suisse.

Résultats

Obama nungara a été signalé de 72 des 96 départements de France métropolitaine. L'espèce est particulièrement abondante le long de la côte atlantique, de la frontière espagnole à la Bretagne, et le long de la côte méditerranéenne, de la frontière espagnole à la frontière italienne. Plus de la moitié des signalements provenaient d'une altitude inférieure à 50 m et aucun signalement ne dépassait 500 m ; les régions montagneuses telles que les Alpes, les Pyrénées et le Massif Central ne sont pas envahies. L'abondance locale peut être impressionnante, avec des centaines de spécimens trouvés dans un petit jardin. Une analyse de nos nouvelles séquences de COI, combinée avec des séquences publiées de spécimens de plusieurs pays, a confirmé que trois clades composent l'espèce. Le premier clade, « Brésil », est actuellement confiné à ce pays d'Amérique du Sud ; le deuxième clade, « Argentine 2 », a été trouvé en Argentine et en Europe, uniquement en Espagne ; et le troisième, « Argentine 1 », a été trouvé en Argentine et en Europe, en Espagne, au Portugal, en France, au Royaume-Uni, en Italie, en Belgique et en Suisse. Cela suggère que deux clades d'*O. nungara* d'Argentine ont envahi l'Europe, dont un est largement répandu.

Discussion

Les résultats de ce travail suggèrent fortement qu'*O. nungara* est une espèce hautement envahissante et que la population qui a envahi plusieurs pays d'Europe vient d'Argentine. La grande dispersion de l'espèce et son abondance locale, combinée à son caractère prédateur, font d'*O. nungara* une menace potentielle pour la biodiversité et l'écologie de la faune autochtone du sol en Europe. *Obama nungara* est probablement la plus menaçante de toutes les espèces exotiques envahissantes de Plathelminthes terrestres présentes en Europe.

Introduction

Plusieurs Plathelminthes terrestres ont été signalés comme espèces exotiques envahissantes en Europe (Tableau 1). Chacune de ces espèces présente des caractéristiques distinctes. Certaines sont remarquables par leur taille considérable, comme *Bipalium kewense* et *Diversibipalium multilineatum*, qui peuvent atteindre 30 cm de longueur (Justine et al. 2018b). Certaines n'ont envahi que certaines parties de l'Europe, comme *Arthurdendyus triangulatus*, principalement confiné à la partie nord des îles britanniques (Jones et al. 2001). Certaines sont relativement rares, comme *Caenoplana coerulea* (Justine et al. 2014a), ou peu visibles, comme la petite espèce *Marionfyfea adventor* (Jones & Sluys 2016). Une seule espèce, *A. triangulatus*, a été ajoutée, très récemment, à la liste des espèces exotiques envahissantes préoccupantes pour l'Union européenne (European Union 2019).

Cet article se concentre sur une espèce, *Obama nungara*, qui a déjà été répertoriée dans plusieurs pays européens (Carbayo et al. 2016). Jusqu'à présent, il existe des signalements publiés de l'île de Guernesey, du Royaume-Uni, d'Espagne, du Portugal, de la France, de la Belgique et de l'Italie, des signalements non publiés mais valides d'Irlande et de l'île de Madère, et un signalement présumé des Pays-Bas (Tableau 2). Dans cet article, nous ajoutons un signalement de la Suisse. Aucun signalement n'est connu d'Allemagne (Sluys 2019), ni d'aucun pays à l'est de l'Allemagne.

La recherche sur les espèces envahissantes nécessite la participation du public afin d'obtenir des informations détaillées. Comme nos précédentes études sur les vers plats terrestres envahissants (Justine et al. 2014a, 2014b, 2015, 2018b), cette recherche sur *O. nungara* est basée sur des centaines de signalements provenant d'un grand nombre de citoyens. Nous reconnaissons l'immense valeur de ces documents issus de la science participative (Kullenberg & Kasperowski 2016) ; cependant, dans cet article, nous soulignons certaines limites

concernant l'exactitude des signalements de la science participative en raison du biais associé au buzz médiatique.

L'identification des Plathelminthes terrestres est difficile, et le nom *O. nungara* lui-même n'a été attribué à l'espèce que récemment, en 2016 (Carbayo et al. 2016). Dans cet article, en plus des informations basées sur des données issues de la science participative, nous avons séquencé un certain nombre de spécimens de différentes régions de France pour obtenir des codes-barres COI, et analysé nos séquences à la lumière des recherches récentes sur l'origine géographique de cette espèce envahissante (Lago-Barcia et al. 2019).

Les résultats de cette analyse suggèrent que les spécimens trouvés en France, en Italie et en Suisse, et séquencés par nos soins, ont leur origine géographique en Argentine.

Matériels et méthodes

Science participative et collecte d'informations

Les données ont été collectées de mi-2013 à fin 2018, soit une période de 5 ans et demi. Pour cette étude, nous avons utilisé la même méthode que pour nos recherches antérieures sur les vers plats invasifs (Justine et al. 2014a, 2014b, 2015, 2018b). En bref, un blog (Justine 2019b) et un compte Twitter (https ://twitter.com/Plathelminthe4) ont contribué à la collecte et à la transmission des informations. Des rapports d'observations de Plathelminthes terrestres ont été reçus du grand public et parfois de professionnels, généralement par email et, depuis 2018, grâce à une page web dédiée (http ://eee.mnhn.fr/). Les signalements inexacts (limaces, vers de terre, sangsues et autres) ont été éliminés. Deux vagues de signalements ont été reçues en 2014 et 2015 lorsque nous avons publié nos articles sur *Platydemus manokwari* (Justine et al. 2014b, 2015) et lorsque les médias ont rapporté la présence de cette espèce hautement envahissante en France métropolitaine. De nombreux signalements ont été étiquetés comme

"*Platydemus manokwari*" ou "Ver plat de Nouvelle-Guinée" par le public ; aucun n'était réellement P. *manokwari* à l'état sauvage en France métropolitaine et, dans la plupart des cas, il s'agissait en fait d'*O. nungara*.

Les photographies reçues des citoyens participants ont été étudiées. *Obama nungara* mesure généralement de 5 à 8 centimètres de long, avec une face dorsale brunâtre (variant selon les spécimens de l'orange au presque noir), une face ventrale beige et un corps portant une tête pointue. En France métropolitaine, la confusion sur la base de photographies envoyées par des amateurs n'est possible qu'avec : (1) *Parakontikia ventrolineata*, mais cette espèce est plus petite et présente des lignes longitudinales typiques sur le dos et le ventre ; ces lignes sont souvent peu visibles sur les photographies prises par des amateurs mais peuvent être vues après avoir amélioré le contraste, et (2) *P. manokwari*, mais cette espèce a une forme de corps typique, deux yeux bien visibles et une ligne longitudinale claire et pâle sur son dos. Inversement, *O. nungara* ne peut être confondu avec *Caenoplana bicolor* (face dorsale jaune-orange vif typique avec deux lignes longitudinales noires), *Caenoplana coerulea* (ligne blanche bien visible typique sur le dos, face ventrale bleue) et les bipaliinés (forme de la tête typique, et grande taille pour *B. kewense* et *D. multilineatum*). Une confusion entre *O. nungara* et les petites espèces, comme *Marionfyfea adventor*, *Rhynchodemus* spp. ou *Microplana* spp. est peu probable. Finalement, nous sommes convaincus que tous les signalements mentionnés dans cet article sont bien *O. nungara*.

Quelques contributeurs non professionnels ont décidé, de leur propre initiative, de faire bien plus que ce qui était demandé et ont tenu le compte du nombre de vers qu'ils tuaient au cours de certaines périodes. Quelques exemples de ces informations sont inclus ici. Bien que nous soyons conscients que ces observations n'ont pas été effectuées par des scientifiques, nous ne

voyons pas pourquoi elles pourraient être erronées, et nous pensons donc qu'elles constituent une contribution précieuse à notre travail scientifique.

Dans notre précédent article sur les bipaliinés (Justine et al. 2018b), après avoir sollicité l'autorisation de citoyens, nous avons fait un effort considérable pour publier toutes les photographies disponibles. Cela a pris du temps, car l'autorisation formelle a dû être collectée parfois jusqu'à 5 ans après la réception d'un e-mail. Dans cet article, nous avons reçu un nombre beaucoup plus élevé de signalements de science participative que dans notre article précédent sur les bipaliinés (530 signalements contre 117). Nous avons décidé que la publication de toutes les informations et données photographiques rendrait les fichiers supplémentaires trop volumineux et n'était probablement pas très bénéfique. Au lieu de cela, nous avons sélectionné les photos les meilleures et les plus informatives.

Notre objectif était à l'origine de collecter des informations pour la France métropolitaine, mais quelques signalements ont également été obtenus des pays voisins, à savoir la Suisse et l'Italie.

Dans une perspective de science ouverte, nous rendons publics les 530 signalements obtenus en France, en tant que Fichier supplémentaire 1 ; les signalements ont été anonymisés, c'est-à-dire que les noms des personnes ont été supprimés. Les données seront également transférées vers des bases de données nationales sur la biodiversité, puis vers le Fonds mondial d'information sur la biodiversité (GBIF).

Collectes des spécimens

Dans certains cas, après avoir examiné une photographie, nous avons sollicité des spécimens auprès de citoyens non-professionnels ou de professionnels ayant signalé des observations. Les spécimens ont été envoyés vivants ou dans l'éthanol, enregistrés dans les collections du Muséum National d'Histoire Naturelle, Paris (MNHN), et traités pour analyse moléculaire.

Lorsque les échantillons ont été obtenus vivants, ils ont été fixés dans de l'eau presque bouillante et conservés dans de l'éthanol à 95%.

Séquences moléculaires

Pour l'analyse moléculaire, un petit morceau du corps (1 à 3 mm^3) a été prélevé sur le bord latéral d'individus fixés à l'éthanol. L'ADN génomique a été extrait à l'aide du kit QIAamp DNA Mini (Qiagen). Deux jeux d'amorces ont été utilisés pour amplifier le gène COI. Un fragment de 424 paires de bases (désigné dans ce texte comme "séquence courte") a été amplifié avec les amorces JB3 (= COI-ASmit1) (sens 5′-TTTTTTGGGCATCCTGAGGTTTAT-3′) et JB4.5 (= COI-ASmit2) (anti-sens 5′-TAAAGAAAGAACATAATGAAAATG-3′) (Bowles et al. 1995 ; Littlewood et al. 1997). Les réactions de PCR ont été effectuées dans 20 µl, contenant 1 ng d'ADN, 1 x tampon PCR CoralLoad, 3 mM de $MgCl_2$, 66 µM de chaque dNTP, 0,15 µM de chaque amorce et 0,5 unité d'ADN polymérase Taq (Qiagen). Le protocole d'amplification était : 4 minutes à 94°C, suivi de 40 cycles à 94°C pendant 30 secondes, 48°C pendant 40 secondes, 72°C pendant 50 secondes, avec une extension finale à 72°C pendant 7 minutes. Un fragment de 825 paires de bases a été amplifié avec les amorces BarS (sens 5′-GTTATGCCTGTAATGATTG-3′) (Álvarez-Presas et al. 2011) et COIR (anti-sens 5′-CCWGTYARMCCHCCWAYAGTAAA-3′) (Lázaro et al. 2009 ; Mateos et al. 2013). Les produits de PCR ont été purifiés et séquencés dans les deux sens sur un séquenceur d'ADN 3730xl à 96 capillaires (Applied Biosystems). Les résultats des deux analyses ont été concaténés pour obtenir une séquence COI d'une longueur de 909 pb (désignée dans ce texte comme « séquence longue »). Les séquences ont été vérifiées à l'aide du logiciel CodonCode Aligner (CodonCode Corporation, Dedham, MA, USA), comparées au contenu de la base de données GenBank à l'aide de BLAST, et finalement déposées dans GenBank sous les numéros d'accès

MN529561–MN529582. Pour plusieurs échantillons, seules des séquences « courtes » ont été obtenues (Tableau 3).

Matrice

De nombreuses séquences du gène COI d'*O. nungara* sont disponibles dans GenBank, sous divers noms d'espèces, en raison de la confusion et de l'histoire nomenclaturale complexe de l'espèce (Álvarez-Presas et al. 2014 ; Carbayo et al. 2016 ; Lago-Barcia et al. 2015, 2019). Ces séquences ont été obtenues avec diverses amorces et ne se chevauchent donc que partiellement. Nous avons construit une matrice en trois étapes. Étape 1 : nous avons construit une grande matrice préliminaire comprenant presque toutes les séquences d'*O. nungara* (sous des noms différents) provenant de GenBank et une sélection de séquences d'autres espèces d'*Obama*. Nous avons constaté qu'une partie seulement de la séquence de COI était partagée par un nombre significatif d'entre elles. Étape 2 : nous avons coupé la matrice pour ne conserver que la partie partagée. Les analyses préliminaires avec cette matrice ont produit des arbres avec un faible soutien, car de nombreuses séquences de GenBank avaient des erreurs de lecture. Étape 3 : nous avons supprimé toutes les séquences contenant des erreurs de lecture. La matrice finale, utilisée dans cette analyse, a une longueur de 255 bases et comprend 99 séquences, soit 8 séquences de 7 espèces d'*Obama* (*O. anthropophila*, *O. burmeisteri*, *O. carinata*, *O. josefi*, *O. ladislavii*, *O. marmorata*, *O. maculipunctata*) et 91 séquences d'*O. nungara* dont nos 22 nouvelles séquences. La matrice comprend des séquences d'*O. nungara* d'Argentine, du Brésil et de sept pays d'Europe ; elle est parfaitement « propre » sans erreurs de lecture ou parties vierges, c'est-à-dire qu'elle fournit des informations pour 99 taxons sur les 255 positions.

Dans une perspective de science ouverte, nous rendons cette matrice publique en tant que Fichier supplémentaire 2 avec cet article ; la matrice, au format MEGA (.meg) (Kumar et al. 2016), contient des informations sur les « groupes » (une caractéristique de MEGA), c'est-à-

dire les clades trouvés dans l'analyse. Elle est également fournie au format FASTA (.fasta), qui n'inclut pas ces informations.

Arbres et distances

MEGA7 (Kumar et al. 2016) a été utilisé pour évaluer les distances, choisir des modèles et construire des arbres. Le meilleur modèle évolutif pour l'ensemble de données de notre matrice, sous le critère d'information bayésien (BIC), était le modèle Hasegawa-Kishino-Yano avec distribution gamma (HKY + G) (Hasegawa et al. 1985). L'histoire évolutive a été déduite en utilisant la méthode du maximum de vraisemblance (Maximum Likelihood, ML), avec 1000 réplications de bootstrap. La méthode du voisinage (Neighbour-Joining, NJ) (Saitou & Nei 1987) a également été utilisée à des fins de comparaison, avec 1000 réplications de bootstrap. Les distances entre les clades d'*O. nungara* et à l'intérieur d'entre eux, et les distances entre ces clades et le groupe externe ont été calculées avec diverses méthodes (Tamura-3, distance *p*, Kimura-2 et probabilité composite maximale) (Kimura 1980 ; Tamura 1992 ; Tamura et al. 2004).

Analyses de population

Les réseaux d'haplotypes ont été construits en utilisant la même matrice que pour les arbres, mais limitée à l'espèce *O. nungara*, c'est-à-dire une matrice avec 91 taxons et une longueur de 255 bases. Nous avons utilisé le logiciel PopART (Leigh & Bryant 2015). Dans le fichier nexus, les caractères étaient l'origine géographique du spécimen, avec un nombre de caractères (NTRAITS) défini sur 9 et des étiquettes de caractères définies comme suit : Argentine, Brésil, Royaume-Uni (y compris Guernesey), Portugal, Espagne, Italie, Suisse, Belgique et France métropolitaine. La méthode de Templeton, Crandall et Sing (TCS) a été utilisée pour déduire les relations entre les échantillons (Clement et al. 2002).

Toujours dans une perspective de science ouverte, nous rendons cette matrice publique en tant que Fichier supplémentaire 3, au format Nexus (.nex).

Résultats

Les spécimens adultes et/ou les photographies de spécimens adultes qui nous ont été envoyés correspondaient bien à la description de la morphologie externe et aux photographies publiées d'*O. nungara*, y compris les formes foncées et claires (Carbayo et al.2016). Aucun de nos échantillons n'a été examiné histologiquement.

Morphologie d'*Obama nungara* de France métropolitaine

Au repos, *O. nungara* est aplati et en forme de feuille (Fig. 1), et lorsqu'il est actif, il est allongé avec des marges parallèles se rétrécissant doucement vers l'extrémité antérieure en pointe et se rétrécissant plus brusquement vers l'extrémité postérieure.

Dans les spécimens vivants examinés dans cette étude, la couleur de fond dorsale variait d'un brun miel clair uniforme à un brun foncé, avec des stries et des pointillés brun foncé.

Dans les spécimens de couleur claire (Figs 1, 2), les bandes longitudinales dorsales médianes appariées mal définies de pigment brun foncé sont séparées par une fine zone médiane brun clair de couleur de fond pâle, sans stries ni pointillés, qui s'étend de la pointe antérieure à l'extrémité postérieure. Les marges médianes de ces rayures sont nettes et les marges extérieures diffuses et inégales. Le pigment foncé des bandes médianes appariées est concentré sur la région du pharynx et des organes copulateurs (Fig. 1) dans le tiers postérieur du corps. Au milieu du corps, les larges barres transversales pigmentées d'environ un tiers de la largeur du corps sont séparées par des intervalles étroits de couleur de fond de chaque côté de la ligne

médiane pâle (Fig. 2). Chez certains spécimens, ces taches de pigmentation ressemblent à des « rayures tigrées » et peuvent continuer en avant vers la pointe (Fig. 3).

À l'extérieur de ces « bandes tigrées » médianes appariées, des stries brunes composées d'agrégations de pigment foncé sont orientées longitudinalement le long du corps, et leur couleur et motif donnent au corps une fine texture gneissique. Une zone submarginale de couleur de fond claire, dépourvue de stries ou de pointillés, borde tout le corps presque jusqu'à la pointe antérieure brun foncé.

La surface ventrale est de couleur gris-crème pâle (beige pâle), plus claire au milieu (Fig. 4).

Une seule rangée d'yeux fait le tour de la pointe antérieure, se rassemble légèrement antéro-latéralement, puis se propage dorsalement, couvrant environ un tiers de la largeur du corps (Fig. 5). Les yeux, en particulier ceux situés derrière la pointe antérieure et ceux qui s'étendent dorsalement, peuvent présenter une zone dépourvue de pigment entourant chaque œil, ressemblant à de petits halos, qui sont plus visibles chez les individus sombres que chez les clairs.

La forme brun foncé (Fig. 6) était la variété de couleur la plus abondante des spécimens examinés dans cette étude. Chez les spécimens de couleur brun foncé, la bande longitudinale médiane pâle peut presque être effacée par les bandes médianes pigmentées foncées denses ; la pointe antérieure est d'un brun clair et les yeux, derrière la pointe antérieure et dorsalement, présentent un « halo » (Fig. 7).

Les spécimens adultes vivants étaient de taille moyenne, variant en longueur de 52 mm à 108 mm avec par exemple un individu mesurant 68,0 mm de long, de largeur maximale 5,5 mm, avec la bouche à 41,5 mm (61%) et le gonopore à 53 mm (77,9%) en arrière de la pointe antérieure (mesuré sur une photographie).

Un certain nombre d'échantillons conservés et séquencés ont été mesurés ; les mesures sont données en mm, comme la longueur (L), la distance de la bouche de la pointe antérieure (G), la distance du gonopore de la pointe antérieure (G), et les pourcentages : MNHN JL092A, L 40, M 25 (62,5%), G 33 (82,5%) ; MNHN JL094, L 28, M 15 (53,6%), G 20 (71,4%) ; MNHN JL101A, L 40, M 25 (62,5%), G 33 (82,5%) ; MNHN JL101B, L 48, M 28 (58,3%), G 38 (79,2%).

Les cocons étaient rouge vif lorsqu'ils étaient fraîchement pondus (Fig. 8), et passent au brun foncé en 3 jours (Fig. 9). Les cocons ont mis de 13 à 17 jours à éclore à une température d'environ 20°C. Trois juvéniles fraîchement émergés de leur cocon, pondus par le spécimen MNHN JL092 (Fig.10), mesuraient 10 × 1,4 mm, 7 × 1,1 mm et 4,1 × 1,3 mm. Ils étaient de couleur crème clair avec de fines pointes brun foncé, avec une certaine agrégation des taches fines vers le milieu du dos, délimitant une fine bande dorsale médiane discontinue non pigmentée, et un soupçon de barres transversales sombres. Les extrémités antérieures étaient gris foncé et les surfaces ventrales gris pâle.

Comme expliqué précédemment, nous avons reçu des centaines de photographies de spécimens adultes prises par des contributeurs non professionnels et ne pouvons pas montrer toutes les images ici. Cependant, la plupart peuvent être récupérés à partir du fil Twitter de l'un des auteurs (@Plathelminthe4). Nous ne montrons ici que deux photographies d'un intérêt particulier. La figure 11 est un spécimen qui montre le motif brun foncé ; ce spécimen, unique, a été trouvé à Paris en décembre 2013, et cette photo a donc été utilisée par de nombreux médias ; le spécimen a été déposé dans notre collection sous le numéro MNHN JL094 et séquencé (Tableau 3). La figure 12 est une photographie d'une boîte en plastique avec sa surface recouverte de spécimens adultes, qui illustre l'abondance de l'espèce dans certains jardins. La figure 13, avec un échantillon de 9 photographies, montre la variété des images d'*O. nungara*

reçues de divers contributeurs, avec et sans échelle, parfois dans l'habitat naturel ou, au contraire, disposé sur une surface colorée par les photographes.

Information obtenue par la science participative : présence

Notre base de données des signalements valides de vers plats terrestres reçus de la mi-2013 à décembre 2018 contient plus de 1000 signalements, après élimination des espèces qui ne sont pas des vers plats. Nous avons constaté que 530 signalements valables, soit plus de la moitié d'entre eux, concernaient *O. nungara* en France métropolitaine. Il s'agit donc de l'espèce la plus souvent signalée. La plupart des signalements provenaient de non-professionnels, mais quelques-uns provenaient de professionnels ou de scientifiques.

La figure 14 est une carte de ces signalements en France métropolitaine, chaque signalement étant représenté par un point sur la carte. La carte comprend des informations générales codées par couleur sur l'altitude. Les signalements étaient les plus abondants le long des côtes atlantique et méditerranéenne ; les zones montagneuses (Alpes, Pyrénées, Massif Central) étaient dépourvues de signalements, qui étaient principalement dans les plaines. Le quart nord-est du pays, qui a un climat plus froid, avait moins de signalements que les trois autres quarts ; cependant, il y avait une concentration de signalements autour de Paris.

Dans la figure 14, un certain nombre de signalements peuvent être ignorés lorsque des points sont superposés, ce qui arrive quand plusieurs signalements ont été reçus de la même localité ou de localités voisines. La figure 15 est une carte de la France métropolitaine avec les signalements comptés par département (les départements sont les principales divisions administratives en France, avec un nombre actuel de 96 pour la France métropolitaine). Parmi les 96 départements, 72 (75%) avaient des signalements. Les départements avec le plus grand nombre de signalements étaient la Gironde et le Finistère (40 à 80 signalements), sur la côte

atlantique, et la plupart des départements le long de la côte atlantique de la frontière espagnole à la Bretagne avaient plus de 30 signalements. Un cas notable est la Haute-Garonne, qui n'est pas sur la côte mais se démarque des départements voisins avec plus de 40 signalements. Seuls 24 départements n'avaient aucun signalement ; ils se trouvent principalement dans le Massif Central et le quart nord-est du pays. Dans la discussion, nous commentons brièvement ces résultats au vu des densités de population dans les différents départements.

Nous avons utilisé notre base de données pour étudier l’influence de l’altitude. La figure 16 montre un graphique du nombre de signalements en fonction de l’augmentation de l’altitude. Plus de la moitié des signalements provenaient d’une altitude inférieure à 50 m ; les mentions au-dessus de 250 m étaient rares et aucun signalement ne dépassait 500 m.

Information obtenue par la science participative : abondance

En plus des nombreux signalements « ponctuels » mentionnés ci-dessus, nous avons reçu deux rapports de non-professionnels qui ont compté les vers plats dans leurs jardins sur une longue période.

Sylvain Petiet, de Cabanac-et-Villagrains (Gironde, sud-ouest de la France), a collecté et détruit des spécimens chaque jour dans son jardin de mai à août 2014 ; il a estimé la partie envahie à environ 300 m^2, dans un jardin d’environ 5000 m^2 de surface. Il a collecté un total de 924 spécimens : du 3 mai 2014 au 29 mai 2014 (26 jours), 180 spécimens (6,9/jour), et du 29 mai 2014 au 22 août 2014 (85 jours), 744 spécimens (8,7/jour). Après cela, il a rapporté en 2014 que les vers plats étaient toujours aussi nombreux dans le jardin. La figure 13 est une photographie d’une boîte de vers collectée en mai 2014 par Sylvain Petiet. Fait intéressant, il a signalé en 2019 qu'aucun ver plat n'était présent.

Michel Hir, de Brest (Finistère, nord-ouest de la France), a collecté et détruit des spécimens chaque jour dans son jardin de novembre 2015 à mai 2016 (Tableau 4). Son jardin fait 175 m^2 de surface. Il a mentionné que le jardin était entouré de murs de 2 mètres de haut et a observé que les spécimens étaient visibles sur le mur mais jamais à plus de 40 cm de hauteur. Nous pouvons donc considérer que tous les spécimens trouvés provenaient de la reproduction des individus de son propre jardin, sans invasion des jardins voisins. Il a collecté un total de 1442 spécimens (10,2/jour). Après cela, il a rapporté que les vers plats étaient toujours aussi nombreux dans le jardin.

Un simple calcul à partir des données de Michel Hir pour la période la plus élevée qu'il a enregistrée (novembre : 19 vers/jour/175 m^2) fournit une estimation de l'augmentation de la population due à la reproduction de 1085 vers/hectare/jour, soit 0,1 ver/m^2/jour. De plus, tous ces dénombrements sont basés seulement sur des individus adultes, ce qui suggère que le nombre de nouveau-nés était encore plus élevé (bien que la mortalité des nouveau-nés n'ait pas été évaluée, nous pouvons supposer qu'elle n'est pas nulle).

Arbres et distances

Un arbre (Fig. 17) a été construit à partir d'une matrice construite à partir d'une sélection de séquences de COI d'*O. nungara* de GenBank et de nos propres séquences nouvelles, avec une sélection de séquences d'autres espèces comme groupe externe. La matrice comprenait 99 taxons avec 255 positions et était parfaitement propre, c'est-à-dire sans parties mal lues ou vierges.

Le clade *O. nungara* comprenait 91 séquences et avait un soutien élevé (ML 96%, NJ 99%). En raison de l'histoire nomenclaturale complexe de l'espèce, de nombreux spécimens étiquetés dans GenBank comme « *Obama marmorata* », et d'autres étiquetés comme « *Obama* sp. » ou

« *Obama* sp. 6 », ont été inclus dans le clade *O. nungara* – nous considérons ces séquences comme des représentants d'*O. nungara*.

Au sein du clade *O. nungara*, il y avait trois clades, chacun avec un soutien élevé. Ces trois clades sont décrits dans l'arbre simplifié de la figure 18. Nous avons choisi les noms des clades en fonction du pays d'origine des séquences d'Amérique du Sud.

Le premier clade (appelé ici clade « Argentine 1 », ML 89%, NJ 91%) est robuste et comprend la plupart des séquences d'*O. nungara*, y compris des spécimens d'Argentine et la plupart des spécimens de pays européens, dont tous les spécimens de France. Ce clade « Argentine 1 » est le groupe-frère d'un clade (à faible soutien) qui comprend deux clades à haut soutien, un clade (ici appelé clade « Argentine 2 », ML 87%, NJ 86%) qui comprend des spécimens provenant exclusivement d'Argentine et Espagne, et un clade (ci-après dénommé clade « Brésil », ML 98%, NJ 98%) qui comprend exclusivement des spécimens du Brésil.

Les distances entre et à l'intérieur des clades qui constituent le clade *O. nungara* sont détaillées dans le Tableau 5. Les distances moyennes à l'intérieur des clades étaient faibles, allant de 0,39 à 1,18% selon le clade et la méthode utilisée, et les distances moyennes entre les clades étaient de 3,62 à 5,39%, selon le clade et la méthode utilisés. Les distances moyennes entre les trois clades d'*O. nungara* et le groupe externe étaient plus élevées, allant de 9,25 à 17,61%, selon le clade et la méthode utilisés. Cela suggère qu'*O. nungara* est composé de trois clades distincts, chacun avec une variation interne relativement faible, bien que présente.

Réseau d'haplotypes

La matrice pour le réseau comprenait 91 séquences d'*O. nungara* de 9 pays ; la matrice avait 255 positions et était propre, sans erreur de lecture ou parties vierges. Le réseau d'haplotypes

est illustré à la figure 19. L'analyse du réseau d'haplotypes a identifié 19 haplotypes pour *O. nungara*. Trois réseaux principaux ont été obtenus.

Le réseau le plus important en termes de nombre de séquences comprenait 10 haplotypes, dont trois étaient représentés par plus de 10 échantillons et les 6 autres étaient soit des singletons (5), soit deux échantillons (1). Ce vaste réseau comprenait des spécimens d'Argentine et de sept pays européens, dont l'Espagne, le Portugal, la France, la Belgique, le Royaume-Uni, l'Italie et la Suisse ; il correspondait au clade « Argentine 1 » de l'arbre. Les trois haplotypes principaux de ce réseau avaient des compositions géographiques différentes. Le plus grand haplotype comprenait des représentants de l'Argentine et de 6 pays européens, le second avait des spécimens d'Argentine et 4 pays européens, et le troisième n'avait que des spécimens d'Espagne ; parmi les petits groupes, 5 viennent d'Espagne et 2 de France.

Un autre réseau comprenait 5 haplotypes, chacun avec 1-2 spécimens, et correspondait au clade « Argentine 2 » de l'arbre ; les spécimens provenaient uniquement d'Argentine (4 haplotypes) et d'Espagne.

Un réseau, avec 4 haplotypes, ne comprenait que des spécimens du Brésil et coïncidait avec le clade « Brésil » trouvé dans l'arbre.

Enfin, la plupart des spécimens d'Europe appartenaient au clade « Argentine 1 », avec seulement quelques spécimens d'Espagne dans le clade « Argentine 2 » ; aucun spécimen d'Europe n'a été trouvé dans le clade « Brésil ».

Les signalements de la science participative et leur signification

Dans la figure 20, nous rapportons le nombre de signalements d'*O. nungara* en France métropolitaine chaque année de 2013 à 2018. Deux pics sont visibles, en 2014 et 2018.

Dans la figure 21, nous montrons les résultats pour *O. nungara* en France métropolitaine classés par mois, pour tous nos signalements de 2013 à 2018. L'histogramme montre une tendance à un pic général à la fin du printemps (mai et juin) et un autre pic, plus faible, en automne (octobre) ; les signalements en hiver sont peu nombreux.

Dans notre travail quotidien de validation et de compilation des signalements de science participative, notre attention a été attirée sur le fait que les mentions de vers plats dans les médias ont été immédiatement suivies par l'arrivée de nombreux signalements, alternant avec des périodes plus calmes quand aucun reportage n'était publié par les médias. Dans la figure 22, nous montrons la variation des signalements hebdomadaires en 2018 pour toutes les espèces de vers plats terrestres, y compris *O. nungara* (qui représente environ la moitié des signalements). Le pic des semaines 21-24 en 2018 coïncide avec la publication de notre article dans PeerJ sur les bipaliinés, qui a bénéficié d'un communiqué de presse et a suscité un vif intérêt de la part des médias en France. Ces observations sont commentées dans la discussion.

Discussion

Populations d'*Obama nungara* et invasion de l'Europe

Nos résultats confirment les conclusions de Lago-Barcia et al. (2019) sur les voies d'invasion d'*O. nungara*. Dans le continent d'origine, l'Amérique du Sud, il existe trois populations connues d'*O. nungara*, deux en Argentine, à savoir « Argentine 1 » et « Argentine 2 » et une au Brésil, à savoir « Brésil ». Seuls des spécimens avec des séquences identiques ou proches des membres des populations « Argentine 1 » (dans sept pays) et « Argentine 2 » (uniquement en Espagne) ont été trouvés en Europe. L'invasion de plusieurs pays d'Europe a son origine en Argentine, pas au Brésil. Notre analyse des séquences disponibles, à l'heure actuelle, suggère

que la population « Argentine 2 » en Europe est toujours limitée à l'Espagne, tandis que la population « Argentine 1 » est largement présente dans la plupart des pays européens envahis.

Notre analyse confirme les résultats publiés (Lago-Barcia et al. 2019), avec des spécimens provenant de pays supplémentaires. Notre matrice comprend un nombre plus élevé de séquences d'*O. nungara* (91 contre 66). Nous n'avons pas utilisé exactement les mêmes séquences et la même matrice, en raison des limitations détaillées dans les Matériels et méthodes, puisque les séquences de différents auteurs ne se chevauchent pas ; cependant, nous avons trouvé des résultats similaires pour l'origine de l'invasion. Notre matrice contient un nombre inférieur de séquences en provenance d'Argentine, car nous avons éliminé les séquences avec des erreurs de lecture, mais cela n'a pas diminué la valeur de l'interprétation. Les réseaux de notre analyse correspondent bien à ceux de l'étude précédente, avec « Argentine 1 » = « Réseau 3 », « Argentine 2 » = « Réseau 1 » et « Brésil » = « Réseau 2 ». En plus de l'étude précédente, notre étude comprend des séquences d'un plus grand nombre de pays et plusieurs nouvelles séquences de France. Nous avons trouvé un total de 19 haplotypes (contre 21) pour *O. nungara*, dont 10 haplotypes dans le plus grand réseau, « Argentine 1 », contre 11 dans le « Réseau 3 » correspondant dans l'étude précédente. Nos résultats confirment et étendent donc les conclusions de Lago-Barcia et al. (2019).

Pour les études futures sur *O. nungara*, les séquences COI, avec une grande quantité de données disponibles, resteront certainement un choix principal, mais des séquences plus variables seront utiles pour une analyse plus fine de l'invasion.

L'étendue de l'invasion de la France métropolitaine et de l'Europe

Comme le montrent les cartes (Figs 14, 15), *O. nungara* a envahi une grande partie de la France métropolitaine et a été trouvé dans 72 des 96 départements de France métropolitaine. L'espèce

a également été signalée en Europe continentale en Espagne, au Portugal, en Italie, en Belgique, peut-être aux Pays-Bas, en Suisse, ainsi que dans des îles européennes au nord telles que Guernesey, la Grande-Bretagne et l'Irlande, et, en mer Méditerranée, en Corse (Tableau 2), ainsi qu'à Madère dans l'Atlantique Nord. L'espèce est apparemment absente d'Allemagne (Sluys 2019), mais étant donné que plusieurs de nos signalements en France métropolitaine ne sont qu'à quelques kilomètres de la frontière allemande, il est probable que l'espèce sera signalée dans ce pays très bientôt. Aucun signalement n'a été rapporté dans les pays à l'est de l'Allemagne.

De nombreux rapports de nouvelles invasions par diverses espèces de vers plats terrestres sont publiés chaque année (Chaisiri et al. 2019 ; Hu et al. 2019 ; Jones 2019 ; Justine et al. 2018a, 2019 ; Prozorova & Ternovenko 2018 ; Rodríguez-Cabrera & Torres 2019 ; Soors et al. 2019 ; Vardinoyannis & Alexandrakis 2019). Comme pour les autres Plathelminthes terrestres (Sluys 2016), l'origine de l'invasion par *O. nungara* est probablement le commerce international des plantes, car les Plathelminthes adultes et les cocons peuvent facilement voyager dans des plantes en pot. Après une invasion à partir de l'Argentine (probablement avec plusieurs vers) vers un pays inconnu (ou peut-être plusieurs pays) en Europe, l'espèce a ensuite été transmise de pays à pays en Europe par le biais du commerce intra-européen des plantes, et à nouveau à l'intérieur de chaque pays de la même façon.

Plus de la moitié des signalements d'*O. nungara* en France métropolitaine provenaient d'une altitude inférieure à 50 m, les signalements au-dessus de 250 m étaient très rares et il n'y avait aucun signalement au-dessus de 500 m (Fig. 16). Bien que la plupart de ces informations d'altitude puissent sembler redondantes avec les informations géographiques, parce que les localités côtières sont à basse altitude, nous pensons que l'absence d'*O. nungara* au-dessus de 500 m est significative. Nous notons également que la plupart, sinon la totalité, des

signalements dans d'autres pays européens proviennent de localités situées à basse altitude. Fait intéressant, le seul signalement en Suisse, un pays montagneux, est à Genève, avec une altitude (369–458 m) en dessous de la limite trouvée en France. Bien que des études plus détaillées soient probablement nécessaires, nous proposons l'hypothèse que le facteur limitant soit le gel, qui est plus fréquent à des altitudes plus élevées. Cela pourrait être un facteur important limitant l'espèce dans son invasion de l'Europe.

Nos informations sur l'abondance locale de l'espèce, bien que basées uniquement sur des données de non-professionnels, sont impressionnantes. Des centaines de spécimens dans un seul jardin ont été signalés à plusieurs reprises, et les dénombrements de Michel Hir et Sylvain Petiet indiquent qu'une zone envahie peut produire, par reproduction, une estimation de mille individus adultes par jour et par hectare.

Biais géographiques et temporels en science participative : un avertissement

Les données de la science participative ont joué un rôle déterminant dans nos recherches. Il a été démontré que les signalements d'espèces envahissantes sont l'un des principaux domaines de recherche utilisant la science citoyenne (Kullenberg et Kasperowski 2016). Cependant, la qualité des données issues de la science participative nécessite une évaluation (Kosmala et al. 2016), et nous tenons à souligner deux biais dans nos données : géographiques et temporels.

Des biais géographiques se produisent lorsque davantage de signalements sont obtenus dans une zone où davantage de membres du public fournissent des rapports, bien que cette zone n'abrite pas réellement plus de spécimens. Cela est évident sur notre carte (Fig. 14) pour les signalements de la région de Paris. La région parisienne abrite 18% de la population française, avec une densité de population beaucoup plus élevée que celle du reste du pays. Cependant, on pourrait faire valoir que les villes sont plus chaudes que la campagne qui les entoure, offrant

ainsi de meilleures conditions pour l’espèce en hiver. Deux régions à forte densité sur nos cartes (Fig. 15) reflètent probablement des densités de population humaine plus élevées : le département de la Gironde comprend Bordeaux, la 6e plus grande ville française en termes de population, et la Haute-Garonne comprend l’aire urbaine de Toulouse, la 4e plus grande zone urbaine en France en termes de population. Un autre biais possible, qui n'est pas facile à quantifier, est l’écart culturel entre les populations urbaines habituées à utiliser les smartphones et les médias sociaux et les populations rurales, souvent plus âgées, où les gens sont peut-être moins familiers avec la technologie moderne. Nous avons des histoires anecdotiques sur cet écart culturel. Dans de rares cas, nous avons reçu des signalements de vers plats sous forme de photographies imprimées sur papier et envoyées par la poste – cela provenait uniquement des populations rurales, pas des villes. Nous avons également eu des discussions par téléphone sur la façon de demander à un petit-fils d’utiliser son smartphone pour photographier les vers plats trouvés dans le jardin de ses grands-parents – les grands-parents eux-mêmes n'ayant qu'une ligne fixe. De toute évidence, les documents issus de la science citoyenne doivent être pris en considération en tenant compte de ce biais. Les différences culturelles entre les populations urbaines et rurales en France sont probablement relativement mineures par rapport à ce que l’on trouve dans d’autres pays ; une étude récente en Thaïlande a révélé que davantage de signalements de *Platydemus manokwari* avaient été reçus des grandes villes côtières que de l’intérieur du pays (Chaisiri et al. 2019), ce qui devrait, à notre avis, être interprété davantage comme un biais géographique/culturel, plutôt que comme un emplacement privilégié pour l’espèce.

Des biais temporels se produisent lorsque davantage de signalements sont reçus à certaines périodes de l’année, indépendamment de l’abondance réelle de l’espèce. Dans notre travail quotidien de réception de courriels de non-professionnels, nous avons noté que des vagues de signalements étaient reçues quelques jours après certains reportages médiatiques mettant en

vedette nos recherches (radio, télévision ou journaux). L'effet médiatique était visible même si les espèces présentées dans les médias étaient différentes (c'est à dire bipaliinés ou *Platydemus manokwari*, par rapport aux autres vers plats terrestres). En fait, les courriels que nous avons reçus mentionnaient souvent la publication récente des médias qui avait fourni l'information ; cela était encore plus visible lorsque les journaux régionaux étaient impliqués, avec des vagues de signalements provenant de certaines régions seulement. La figure 22 montre que les semaines numéros 22–24 en 2018 ont fourni un nombre élevé de signalements ; clairement, ceci était juste après la publication de notre article sur les bipaliinés (Justine et al. 2018b). Cet article a été accompagné d'un court article en français dans le média à accès libre The Conversation (Justine 2018), lu plus d'un million de fois en une semaine (Justine 2019a), et l'article a été mentionné abondamment dans les médias nationaux (Bardou 2018 ; Morin 2018 ; Vidard 2018) et les médias dans d'autres pays (Gabbatiss 2018 ; Guarino 2018). Cependant, mai est également une période où *O. nungara* est abondant et, dans l'hémisphère Nord, une période où les jardiniers amateurs passent beaucoup de temps dans leur jardin. La figure 21 montre le nombre de signalements de l'espèce chaque mois au cours de l'enquête quinquennale. Bien qu'il soit probablement vrai qu'*O. nungara* est moins abondant en hiver (décembre–février), nous ne savons pas avec certitude s'il s'agit d'une diminution réelle de l'abondance ou si le nombre de mentions est faible simplement parce que c'est une période où les jardiniers passent moins de temps dans leurs jardins. De même, le pic d'abondance en mai et juin est probablement authentique, mais les données étaient biaisées par l'abondance des signalements en mai 2018 mentionnée ci-dessus (Fig. 22).

Ces biais géographiques et temporels pourraient entraver une analyse approfondie des données issues de la science citoyenne ; encore une fois, nous insistons sur le fait que les données de science citoyenne sont d'un extrême intérêt, mais que leur interprétation ne doit jamais négliger ces biais possibles.

Conclusion

Dans cet article, nous avons analysé les données de science participative sur une période de cinq ans et demi et montré qu'*O. nungara* a envahi plus des deux tiers de la France métropolitaine, à l'exception des zones montagneuses. Nous avons également constaté que l'abondance locale dans les jardins peut atteindre des centaines de spécimens et que les spécimens d'un jardin envahi peuvent produire, par reproduction sexuelle, une nombreuse descendance.

Notre étude de codes-barres moléculaires, basée sur des séquences nouvellement acquises et d'autres provenant de GenBank du gène de la cytochrome c oxydase 1, a confirmé que les populations d'*O. nungara* trouvées en Europe sont similaires à deux populations d'Argentine (mais pas du Brésil) (Lago-Barcia et al. 2019). Nous avons également confirmé ou montré qu'une seule population avait envahi l'Espagne, le Portugal, la France, le Royaume-Uni, l'Italie, la Belgique et la Suisse.

Bien que plus de dix espèces de vers plats terrestres aient envahi l'Europe (Tableau 1), aucune autre espèce n'est comparable à *O. nungara* en termes de taille des territoires envahis : l'Europe continentale au Portugal, en Espagne, en France, en Italie, en Belgique et en Suisse, les îles d'Europe telles que la Grande-Bretagne, l'Irlande et Guernesey, ainsi que la Corse en Méditerranée et Madère dans l'Atlantique Nord ; et en termes de densité enregistrée d'animaux dans les zones envahies. Ceci désigne clairement *O. nungara* comme l'espèce invasive la plus importante en Europe parmi les vers plats terrestres.

Étant donné que l'espèce a des habitudes prédatrices sur les espèces de la faune du sol, son impact écologique est probablement élevé, mais n'a pas encore été étudié. De plus, comme l'espèce semble avoir un large spectre de proies dans sa zone d'origine (Boll & Leal-Zanchet

2016), y compris les vers de terre et les mollusques, d'autres études sont nécessaires pour déterminer quelles espèces, et en quelles quantités, sont les plus exploitées en Europe. Des méthodes moléculaires (Cuevas-Caballé et al. 2019) sont disponibles pour obtenir ces informations.

Remerciements

Nous remercions tous les membres du grand public qui ont participé à l'enquête ; ceux qui ont envoyé des spécimens sont particulièrement remerciés. Les noms des non-professionnels, et parfois des scientifiques, qui ont fourni des photographies et/ou des spécimens sont indiqués dans les tableaux et les figures. Deux contributeurs, Sylvain Petiet et Michel Hir, ont aimablement fourni leurs résultats de comptages. Le soutien de différentes Fédérations Régionales de Défense contre les Organismes Nuisibles (FREDON) est reconnu. Hugh Jones (NHM, Londres) a aimablement fourni des informations non publiées sur les signalements au Royaume-Uni, en Irlande et à Madère. Olivier Gargominy (MNHN, Paris) a identifié un escargot à partir de photographies.

Tableau 1. Plathelminthes terrestres envahissants trouvés en Europe, auteurs des taxons et références clés.

Ce tableau fournit des informations complètes sur les auteurs, les taxons et les combinaisons, rendant ainsi le texte général plus léger. Sluys (2016) a répertorié d'autres espèces avec des signalements et des informations limités : *Artioposthia exulans* Dendy, 1901, *Australoplana sanguinea* (Moseley, 1877), *Dolichoplana striata* Moseley, 1877 et *Kontikia andersoni* Jones, 1981. Ce tableau met à jour un tableau similaire publié dans Justine et al. (2018). Les synonymes sont limités aux binômes, les divers « sp. » dans GenBank ne sont pas répertoriés.

Taxon et auteurs	Synonymes	Références pour les taxons	Références principales pour la présence en Europe
Obama nungara Carbayo, Álvarez-Presas, Jones & Riutort, 2016	*Obama marmorata pro parte.*	Carbayo et al. 2016	Carbayo et al. 2016 ; Lago-Barcia et al. 2019 ; cet article
Arthurdendyus triangulatus (Dendy, 1896) Jones, 1999	*Artioposthia triangulata*	Dendy 1895 ; Jones 1999	Boag et al. 1994
Australopacifica atrata (Steel, 1897)	*Geoplana atrata ; Parakontikia atrata ; Kontikia atrata*	Steel 1897	Jones 2019
Bipalium kewense Moseley, 1878		Moseley 1878	Justine et al. 2018b
Caenoplana bicolor (Graff, 1899) Winsor, 1991	*Geoplana bicolor*	von Graff 1899 ; Winsor, 1991	Álvarez-Presas et al. 2014
Caenoplana coerulea Moseley, 1877		Moseley 1877	Álvarez-Presas et al. 2014 ; Breugelmans et al. 2012
Diversibipalium "black"		Justine et al. 2018b	Justine et al. 2018b
Diversibipalium multilineatum (Makino & Shirasawa, 1983) Kubota & Kawakatsu, 2010	*Bipalium multilineatum*	Makino & Shirasawa 1983 ; Kubota & Kawakatsu 2010	Justine et al. 2018b ; Mazza et al. 2016
Marionfyfea adventor Jones & Sluys, 2016		Jones & Sluys 2016	Jones & Sluys 2016
Parakontikia ventrolineata (Dendy, 1892) Winsor, 1991	*Kontikia ventrolineata*	Dendy 1892 ; Winsor 1991	Álvarez-Presas et al. 2014
Platydemus manokwari de Beauchamp, 1963		de Beauchamp 1962	Justine et al. 2014b

Tableau 2. Signalements d'*Obama nungara* en Europe.

L'année indiquée est celle de la date du premier signalement ou du premier spécimen mentionné dans la référence ; la présence réelle de l'espèce peut être antérieure à l'année mentionnée.

Pays ou territoire	Année	Référence
Île de Guernesey	2008	Carbayo et al. 2016
Royaume-Uni	2009	Carbayo et al. 2016
Espagne	2010	Carbayo et al. 2016
Portugal	N/A	Lago-Barcia et al. 2019
France métropolitaine (Corse non comprise)	2013	Justine et al. 2014a ; cet article
Corse	2013	Cet article
Italie	2012	Carbayo et al. 2016 ; cet article
Suisse	2014	Cet article
Irlande	2009	Hugh Jones, communication personnelle, 2019 ; collecte et identification : R. Anderson.
Île de Madère	2018	Hugh Jones, communication personnelle, 2019
Pays-Bas<br>Au Royaume-Uni, dans une plante en pot prétendument « importée des Pays-Bas »	2016	Aldred 2016
Belgique	2017	Soors et al. 2019

Tableau 3. Spécimens d'*O. nungara* utilisés pour l'analyse moléculaire.

Tous les spécimens étaient des adultes, sauf JL067, reçus sous forme de cocons. Tous les signalements proviennent de France, sauf un signalement d'Italie et un de Suisse.

GenBank	MNHN	Date	Localité	Département ou pays	COI	Collecteur
**MN529561**	**JL055**	30/04/2013	Cagnes-sur-Mer	Alpes-Maritimes	Long	Pierre Gros
**MN529562**	**JL057B**	17/05/2013	Lamballe	Côtes-d'Armor	Long	Benoît L'Hotellier
**MN529563**	**JL067**	10/10/2013	Guiclan	Finistère	Long	Madame Stephan (Cocon)
**MN529564**	**JL080**	19/08/2013	Salernes	Var	Long	Daniel Juif
**MN529565**	**JL092A**	26/11/2013	Montauban	Tarn-et-Garonne	Long	Céline Tan
**MN529566**	**JL094**	04/12/2013	Paris	Paris	Long	Xavier Japiot (VILLE PARIS)
**MN529567**	**JL098A**	27/03/2014	Les Matelles	Hérault	Court	Benoit Jaillard (INRA)
**MN529568**	**JL098C**	27/03/2014	Les Matelles	Hérault	Court	Benoit Jaillard (INRA)
**MN529569**	**JL101**	02/04/2014	Hérouville-Saint-Clair	Calvados	Court	Daniel Loisel (FREDON)
**MN529570**	**JL103**	21/06/2014	Ile de Ré	Charente-Maritime	Court	Félix Bécheau
**MN529571**	**JL106**	02/04/2014	Metz	Moselle	Long	Charlie Sommer (FREDON)
**MN529572**	**JL109A**	29/03/2014	Rome	Italie	Long	Di Pompeo et al.
**MN529573**	**JL145**	06/05/2014	La Plaine Saint Denis	Seine-Saint-Denis	Court	Dhyma Gomez
**MN529574**	**JL147A**	16/05/2014	La Flocellière (Sèvremont)	Vendée	Court	Jocelyn Foucher
**MN529575**	**JL152**	24/06/2014	Cagnes-sur-Mer	Alpes-Maritimes	Long	Pierre Gros
**MN529576**	**JL168A**	14/08/2014	Geneva	Suisse	Long	Corinne Jasquelin (DGNP, Genève)
**MN529577**	**JL193**	19/10/2014	Lay-Saint-Christophe	Meurthe-et-Moselle	Long	Emeline Notte (AREXHOR)
**MN529578**	**JL196**	07/11/2014	Châtenay-Malabry	Hauts-de-Seine	Long	Thibault Garnier Boudier (DEPT)
**MN529579**	**JL243**	26/03/2015	Caen	Calvados	Long	Arnaud Pudepiece (FREDON)
**MN529580**	**JL245**	21/03/2015	Brétigny-sur-Orge	Essonne	Long	Nicolas Puillandre (MNHN)
**MN529581**	**JL246A**	07/04/2015	Les Matelles	Hérault	Long	Benoit Jaillard (INRA) et étudiants
**MN529582**	**JL259B**	26/05/2015	Lyon	Rhône	Court	Cloé Laurent (VILLE DE LYON)

Tableau 4. Nombre de spécimens d'*Obama nungara* dans un jardin.

Les spécimens ont été collectés et détruits chaque jour par un non-professionnel dans un jardin de 175 m^2 en France métropolitaine, de novembre 2015 à mai 2016. Mai 2016, seulement les 20 premiers jours. Données de Michel Hir.

	Novembre	Décembre	Janvier	Février	Mars	Avril	Mai (20 jours)	Total
Total	477	414	110	107	52	232	50	1442
Moyenne/jour	19	17	6	6	4	11	8	10

Tableau 5. Distances génétiques entre et à l'intérieur des clades d'*Obama nungara*.

Les résultats sont donnés sous forme de pourcentages et de distances moyennes, selon plusieurs méthodes (Tamura-3, paramètre Kimura-2, distance *p*, vraisemblance composite maximale). Les différences entre les différentes méthodes étaient mineures, et les trois clades d'*O. nungara* étaient bien séparés entre eux et par rapport au groupe externe et présentaient une variation intragroupe mineure (en italique). Le groupe externe était composé de 7 autres espèces d'*Obama*.

**Méthode**			
**Tamura-3**			
	Argentine 1	Argentine 2	Brésil
À l'intérieur du groupe	*0.40*	*1.15*	*0.53*
Argentine 2	3.89		
Brésil	4.32	4.78	
Groupe externe	10.69	11.21	11.49
**distance *p***			
	Argentine 1	Argentine 2	Brésil
À l'intérieur du groupe	*0.39*	*1.12*	*0.52*
Argentine 2	3.62		
Brésil	4.07	4.47	
Groupe externe	9.25	9.64	9.82
**Kimura-2**			
	Argentine 1	Argentine 2	Brésil
À l'intérieur du groupe	*0.40*	*1.15*	*0.53*
Argentine 2	3.84		
Brésil	4.31	4.76	
Groupe externe	10.60	11.11	11.39
**Vraisemblance composite maximale**			
	Argentine 1	Argentine 2	Brésil
À l'intérieur du groupe	*0.40*	*1.18*	*0.54*
Argentine 2	4.11		
Brésil	4.89	5.39	
Groupe externe	15.76	16.89	17.61

Figures

[Note : les figures dans ce fichier ont été amenées à une résolution assez basse – les figures en pleine résolution sont disponibles dans l'article original).

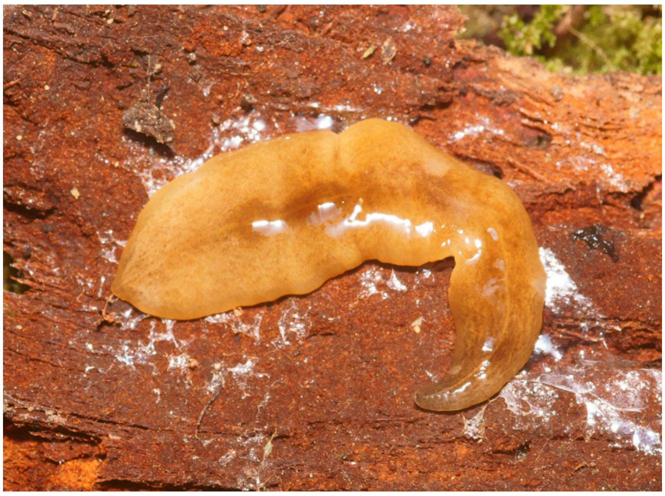

Figure 1. Un exemple de la forme à couleur pâle d'*Obama nungara*, au repos.

Spécimen MNHN JL055 de Cagnes-sur-Mer, Alpes-Maritimes. Photo par Pierre Gros.

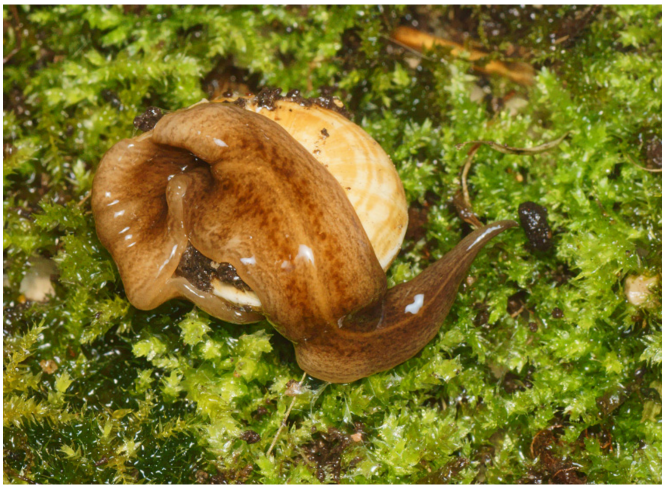

Figure 2. Forme jaune pâle d'*Obama nungara*, se nourrissant d'un escargot.

Le spécimen montre des agrégations transversales de pigment de chaque côté de la bande dorsale médiane pâle. Ce spécimen se nourrit d'un escargot (*Theba pisana*), et le plissement dorsal sur le ver plat résulte de la saillie du pharynx dans l'escargot. Spécimen de Cagnes-sur-Mer, Alpes-Maritimes. Photo par Pierre Gros.

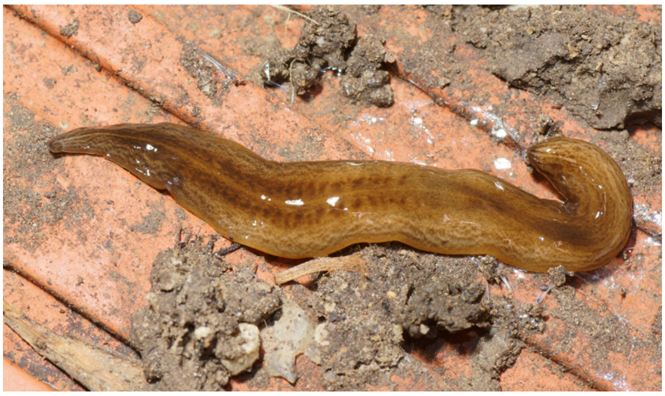

Figure 3. Vue dorsale d'*Obama nungara*.

Ce spécimen présente des « rayures tigrées » dorsales prononcées. Spécimen de Cagnes-sur-Mer, Alpes-Maritimes. Photo par Pierre Gros.

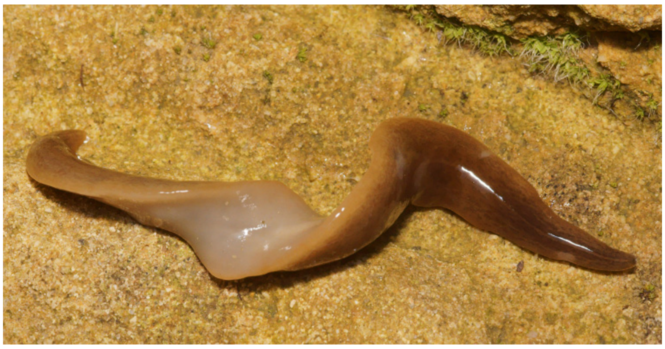

Figure 4. *Obama nungara*, spécimen montrant les faces dorsale et ventrale.

La surface ventrale est pâle. Spécimen MNHN JL055 de Cagnes-sur-Mer, Alpes-Maritimes. Photo par Pierre Gros.

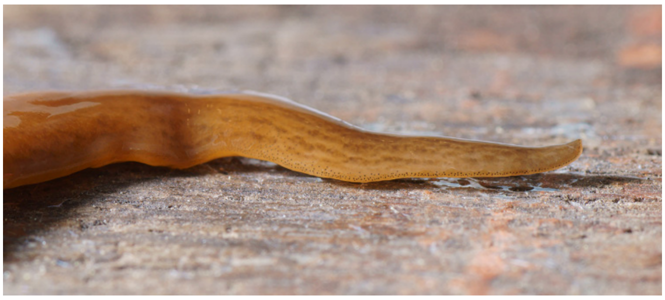

Figure 5. *Obama nungara*, partie antérieure montrant les yeux, vue latérale droite.

L'effet « halo » d'une zone claire autour des yeux est présent, bien que relativement discret chez la forme pâle par rapport à la forme plus foncée. Spécimen MNHN JL055 de Cagnes-sur-Mer, Alpes-Maritimes. Photo par Pierre Gros.

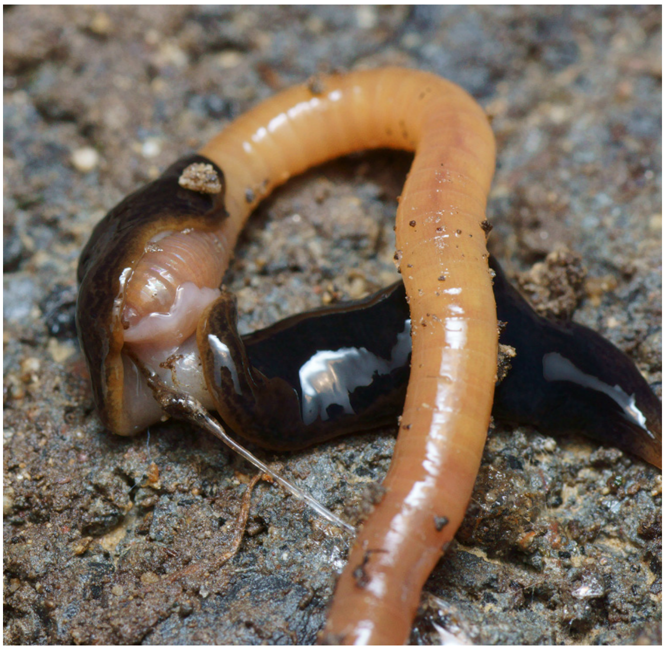

Figure 6. *Obama nungara*, forme sombre se nourrissant d'un ver de terre.

Le pharynx inversé peut être clairement vu enveloppant en partie la tête du ver de terre (espèce non identifiée). Spécimen MNHN JL092 de Montauban, Tarn-et-Garonne. Photo par Pierre Gros.

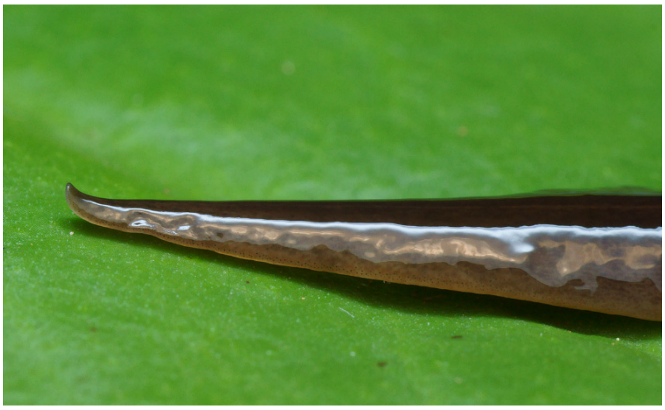

Figure 7. *Obama nungara*, forme sombre, partie antérieure montrant les yeux.

Aspect antérolatéral montrant un « halo » autour des yeux. Spécimen d'Antibes, Alpes-Maritimes. Photo par Pierre Gros.

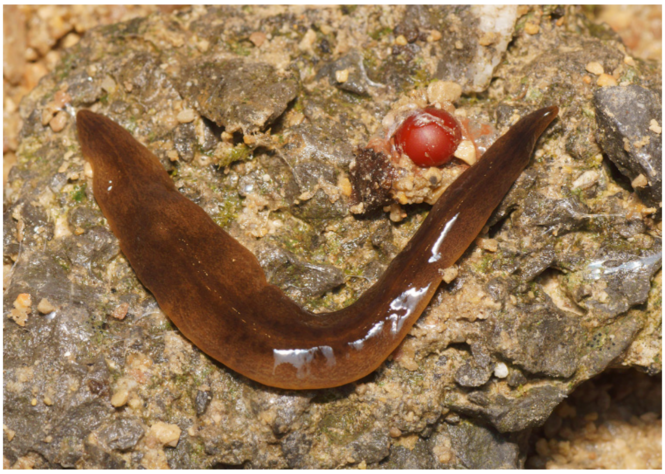

Figure 8. *Obama nungara*, spécimen adulte avec cocon.

Le cocon a été fraîchement pondu et mesure 4,8 mm de diamètre ; sa couleur est rougeâtre. Photo par Pierre Gros.

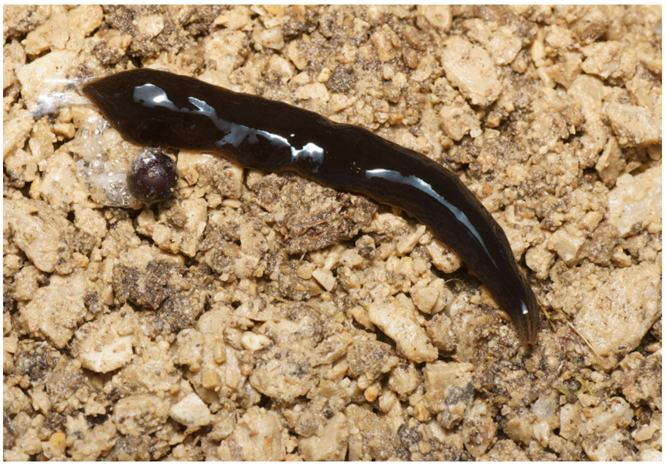

Figure 9. *Obama nungara*, spécimen adulte avec cocon.

Le cocon, 82 heures après sa ponte, mesure 3,7 à 3,9 mm de diamètre ; sa couleur est maintenant noire. Cocon déposé par spécimen MNHN JL92 de Montauban, Tarn-et-Garonne. Photo par Pierre Gros.

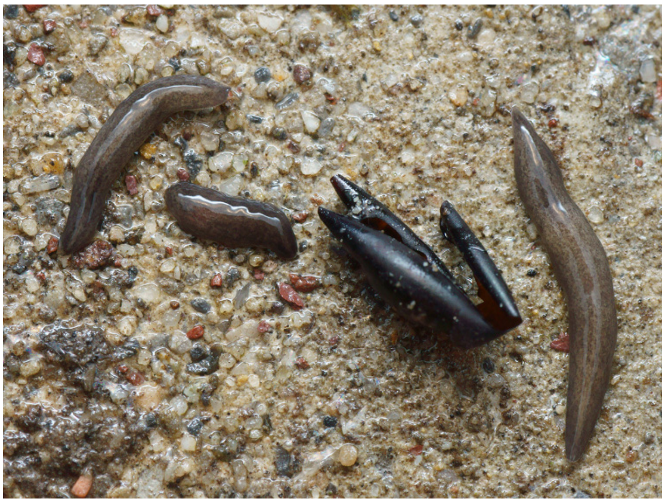

Figure 10. *Obama nungara*, juvéniles.

Le cocon d'oeufs ouvert et les trois juvéniles qu'il contenait. Cocon déposé par le spécimen MNHN JL092 de Montauban, Tarn-et-Garonne. Photo de Pierre Gros.

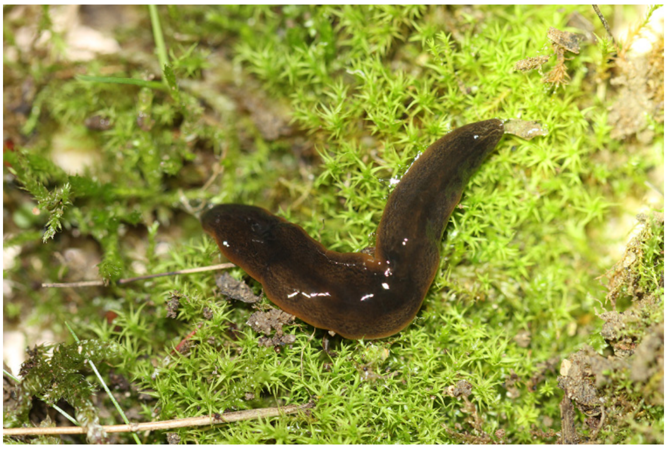

Figure 11. *Obama nungara* trouvé à Paris en décembre 2013.

Ce spécimen unique a été trouvé au Bois de Vincennes, Paris et la photographie a été largement utilisée par la presse française. Spécimen MNHN JL094, code-barres. Photographie par Xavier Japiot, CC-BY-SA-3.0.
https ://commons.wikimedia.org/wiki/File :Planaria_Geoplanidae,_3_cm_(02).JPG

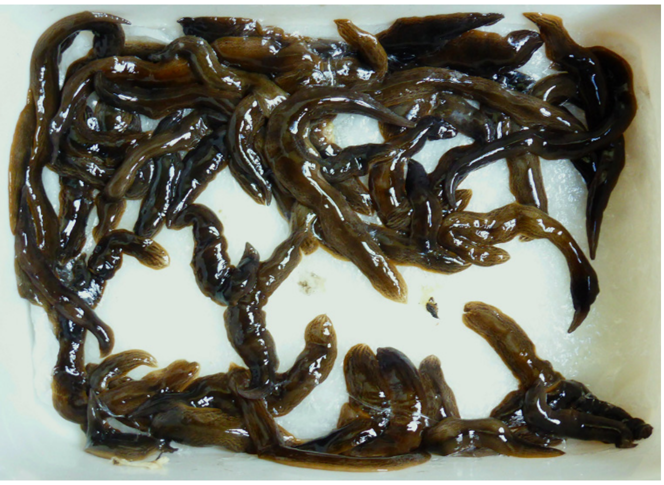

Figure 12. Abondance des spécimens d'*Obama nungara* trouvés dans un jardin.

Une boîte remplie de spécimens adultes collectés à la main par un non-professionnel en une seule journée de mai 2014, dans un petit jardin à Cabanac-et-Villagrains (Gironde). Photographie par Sylvain Petiet.

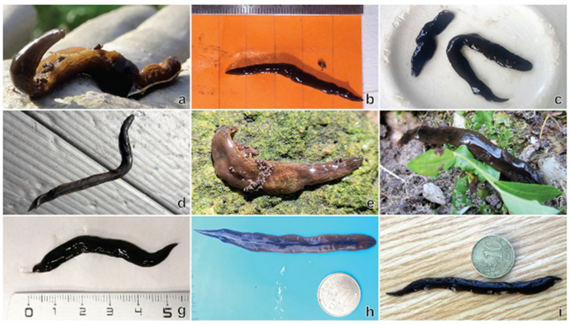

Figure 13. Un échantillon des photographies d'*Obama nungara* dans des jardins, reçues de non-professionnels.

Les photographies en a, e et h sont des exemples de la couleur marron clair ; les autres sont de la forme sombre. Échelles en b, g : centimètres et millimètres ; diamètre de la pièce de 10 centimes d'Euro en h, i : 19,5 mm ; les autres images sont sans échelle.

Tous les auteurs ont accepté de publier leurs photographies sous une licence CC-BY 4.0. a, Cathy Constant-Elissagaray ; b, Nicolas Armengaud ; c, Julien Silvert ; d, Frédéric Madre ; e, Benjamin Klein ; f, Françoise Bronnec ; g, Louise Lejus ; h, Fanny Tourraille ; i, Christophe et Amauray Amiand.

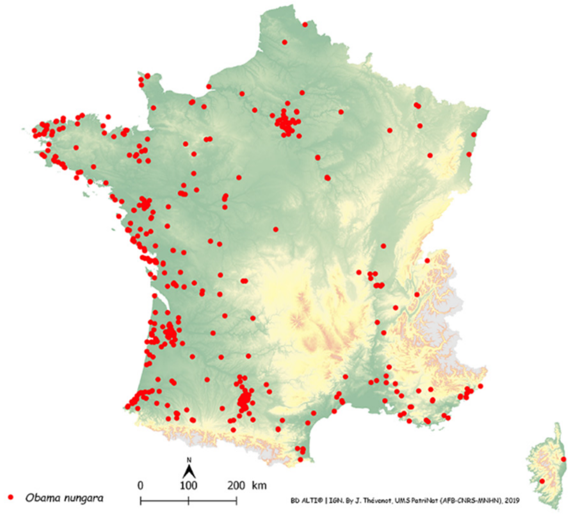

Figure 14. Carte des signalements d'*Obama nungara* en France métropolitaine sur la période 2013-2018.

Chaque point rouge est un signalement pendant la période 2013-2018. Un certain chevauchement entre les points s'est produit. Remarquez la concentration des signalements le long des côtes atlantique et méditerranéenne. Les parties montagneuses (jaunes sur la carte) n'ont pas été envahies.

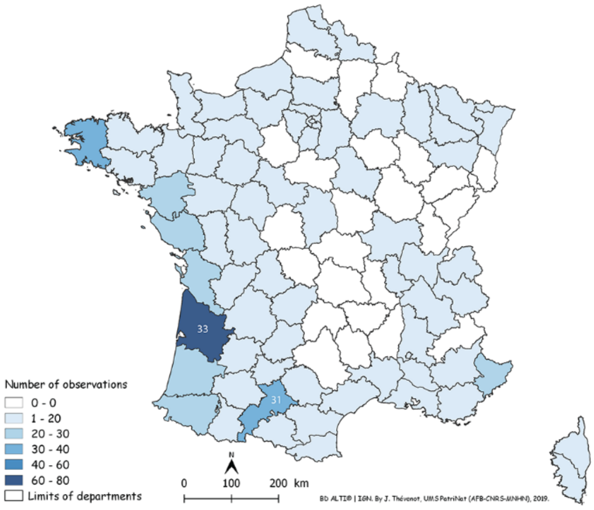

Figure 15. Carte des signalements d'*Obama nungara* en France métropolitaine sur la période 2013-2018, présentés comme nombre de signalements par département.

Les signalements sont indiqués sous forme de nombre de signalements dans chaque département. La couleur montre l'intensité de l'invasion. Notez que tous les départements le long des côtes atlantique et méditerranéenne sont fortement touchés. Les chiffres (31 et 33) montrent deux départements (31, Haute-Garonne ; 33, Gironde) qui sont mentionnés dans la discussion.

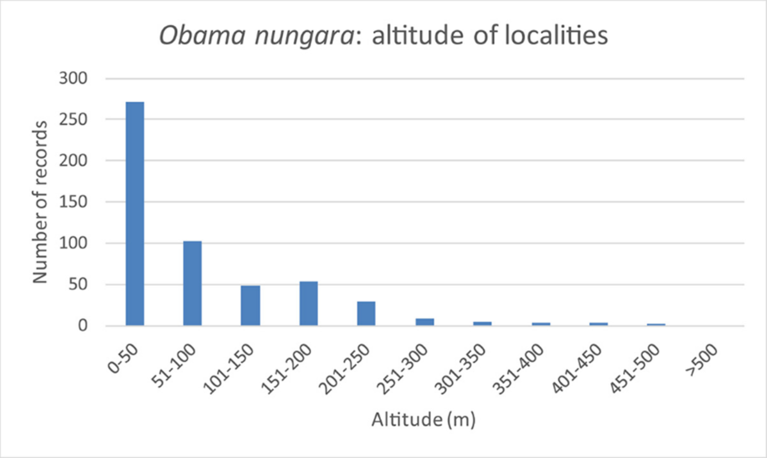

Figure 16. Altitude des localités dans lesquelles *Obama nungara* a été signalé en France métropolitaine.

Plus de la moitié des signalements provenaient d'une altitude inférieure à 50 m et aucun signalement ne dépassait 500 m.

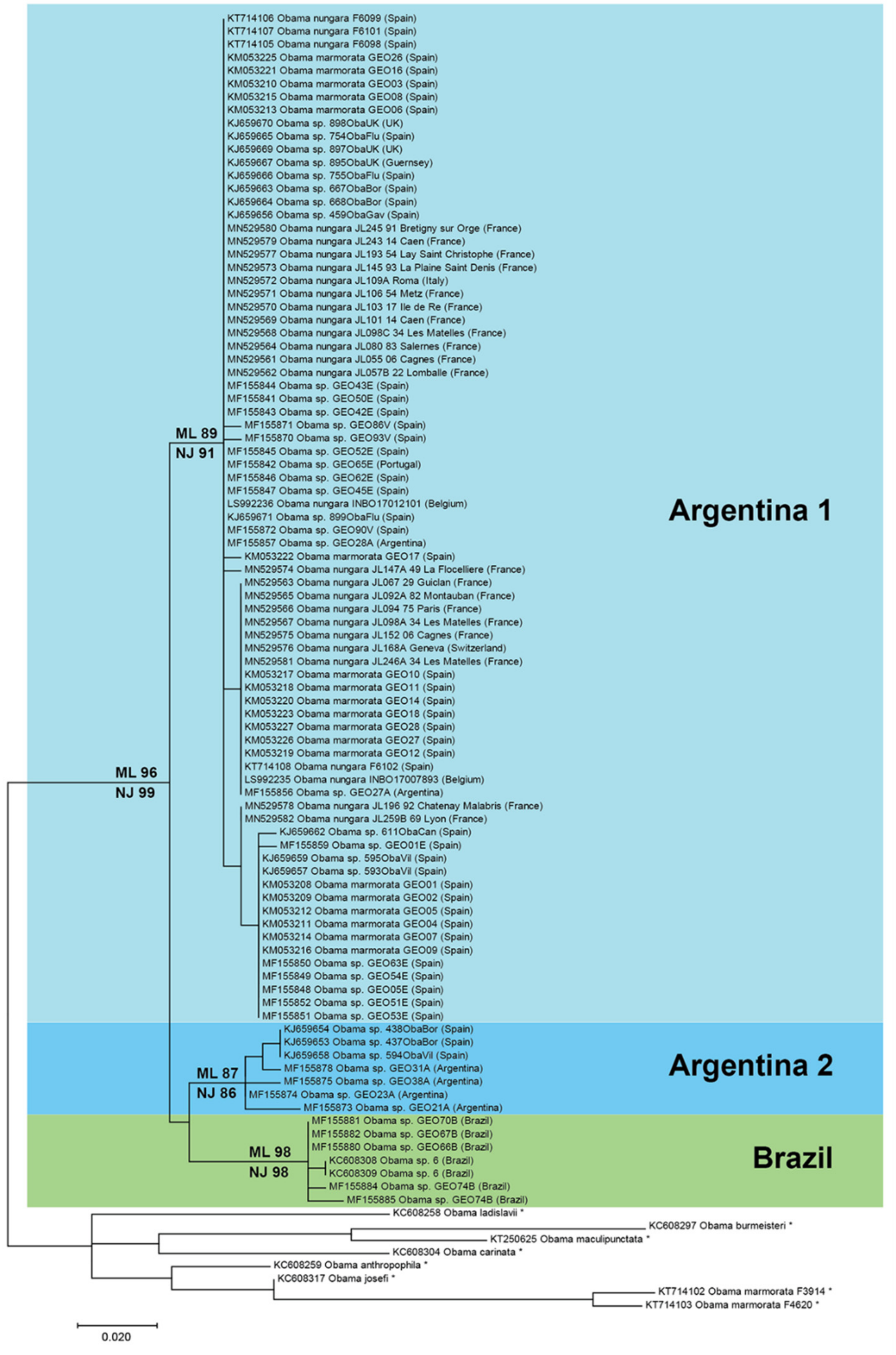

Figure 17. Arbre de maximum de vraisemblance (Maximum Likelihood) d'*Obama nungara* et de ses proches parents.

Les analyses ont impliqué 99 séquences de nucléotides et il y avait un total de 255 positions dans l'ensemble de données final. La matrice comprenait une sélection de séquences d'*O. nungara* disponibles et des séquences de six autres espèces d'*Obama* (notées *), sélectionnées sur l'absence de mauvaise lecture. Le pourcentage d'arbres dans lesquels les taxons associés sont regroupés est indiqué à côté des branches (ML : maximum de vraisemblance ; NJ : voisinage ou Neighbour-Joining). Trois clades sont visibles dans *O. nungara* : « Argentine 1 », « Argentine 2 » et « Brésil », du nom du pays d'origine des séquences sud-américaines. Des

spécimens de diverses localités d'Europe, y compris tous les spécimens de France, ont été inclus dans le clade « Argentine 1 ». Quelques spécimens d'Espagne ont été inclus dans le clade « Argentine 2 ». Aucun spécimen d'Europe n'a été trouvé dans le clade « Brésil ». L'origine géographique est indiquée pour chaque séquence ; pour la France, le numéro d'enregistrement MNHN du spécimen, le numéro de département et la commune administrative sont indiqués. Le clade *O. nungara* comprend de nombreuses séquences qui ont été déposées dans GenBank sous différents noms.

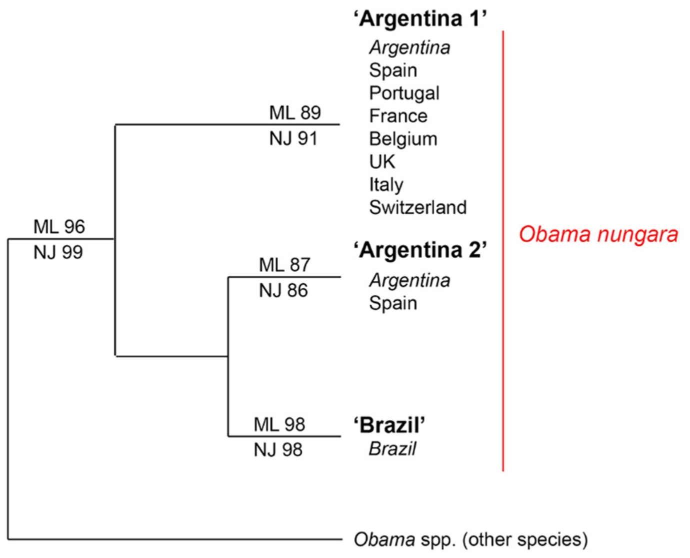

Figure 18. Arbre simplifié des relations au sein des membres d'*Obama nungara* et avec les espèces proches.

Cet arbre simplifié est tiré de l'arbre complet de la figure 17. Il y a trois clades au sein d'*O. nungara*, représentant trois populations différentes au sein de l'espèce : « Argentine 1 », « Argentine 2 » et « Brésil », chacune avec un soutien élevé. Les spécimens invasifs trouvés dans la plupart des pays d'Europe (Espagne, Portugal, France, Royaume-Uni, Italie et Suisse) ont été inclus dans le clade « Argentine 1 » ; cependant, quelques spécimens d'Espagne ont été inclus dans le clade « Argentine ». Aucun spécimen du clade « Brésil » n'a été trouvé en Europe.

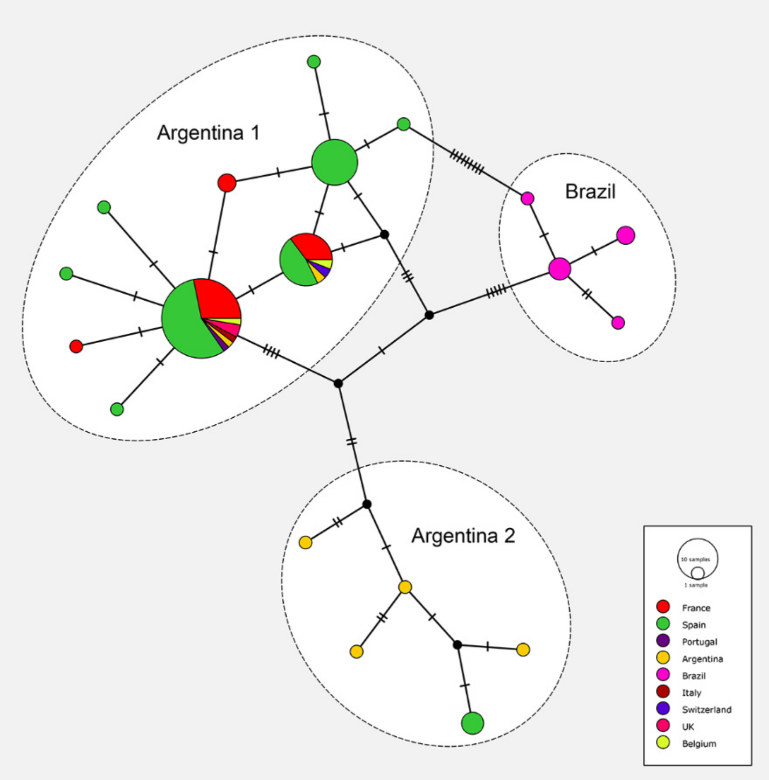

Figure 19. Réseau des relations entre les spécimens d'*Obama nungara* de diverses localités.

Trois groupes sont visibles : « Argentine 1 », « Argentine 2 » et « Brésil ». Le groupe principal, « Argentine 1 », comprend des séquences d'Argentine et de tous les pays envahis en Europe. Le groupe « Argentine 2 » comprend des séquences d'Argentine et quelques-unes d'Espagne, mais pas d'autre pays. Le groupe « Brésil » ne comprend que des spécimens du Brésil, aucun d'Europe. Au sein du groupe « Argentine 1 », trois haplotypes principaux et 7 haplotypes mineurs sont visibles ; deux des principaux haplotypes comprennent à la fois des spécimens d'Argentine et de la plupart des pays européens envahis.

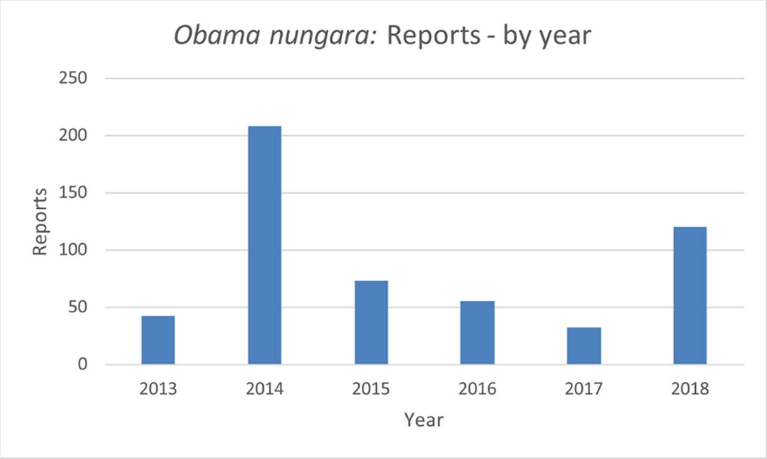

Figure 20. Nombre de signalements d'*Obama nungara* de 2013 à 2018 en France.

Les signalements obtenus par science participative sont indiqués pour chaque année ; 2013 ne comprend que la moitié de l'année. Il y avait un total de 530 signalements vérifiés. Voir le texte pour les commentaires sur les biais probables qui expliquent le nombre plus élevé de signalements en 2014 et 2018.

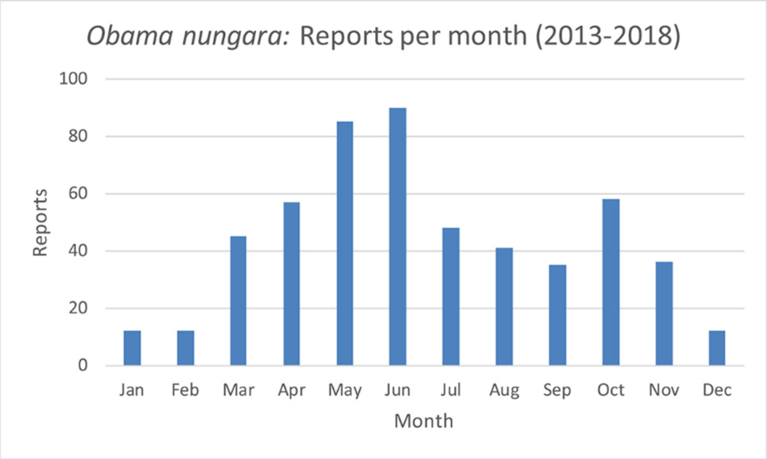

Figure 21. Signalements mensuels d'*Obama nungara* de 2013 à 2018 en France.

Tous les signalements de 2013 à 2018 sont présentés ici sous forme de nombre mensuel de signalements. Le plus grand nombre de signalements a été obtenu en mai-juin. Voir la discussion pour un biais possible pour ces signalements.

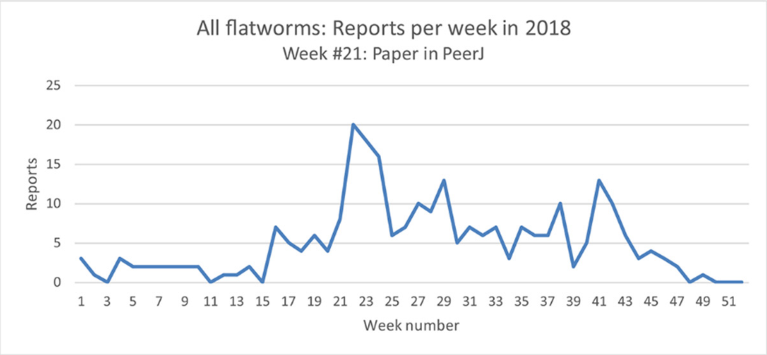

Figure 22. Signalements de Plathelminthes terrestres invasifs en 2018 en France (toutes les espèces, y compris *Obama nungara*), présentés sous forme de chiffres hebdomadaires.

Le nombre total de signalements en 2018 était 262. Le pic des semaines 21-24 a suivi la publication de notre article dans PeerJ, publié le 22 mai 2018, et n'est donc pas une indication d’un nombre plus élevé d’animaux au cours de cette période. Voir la discussion pour les biais temporels dans la science participative.

Références

Aldred J. 2016. Warning over invasive flatworm posing threat to UK wildlife. The Guardian. London, UK.

Álvarez-Presas M, Carbayo F, Rozas J, and Riutort M. 2011. Land planarians (Platyhelminthes) as a model organism for fine-scale phylogeographic studies: understanding patterns of biodiversity in the Brazilian Atlantic Forest hotspot. *Journal of Evolutionary Biology* 24:887-896.

Álvarez-Presas M, Mateos E, Tudo A, Jones H, and Riutort M. 2014. Diversity of introduced terrestrial flatworms in the Iberian Peninsula: a cautionary tale. *PeerJ* 2:e430.

Bardou F. 2018. Comment les vers plats géants ont colonisé les jardins français. Libération. Paris, France.

Boag B, Palmer LF, Neilson R, and Chambers SJ. 1994. Distribution and prevalence of the predatory planarian *Artioposthia triangulata* (Dendy) (Tricladida: Terricola) in Scotland. *Annals of Applied Biology* 124:165-171.

Boll PK, and Leal-Zanchet AM. 2016. Preference for different prey allows the coexistence of several land planarians in areas of the Atlantic Forest. *Zoology* 119:162-168.

Bowles J, Blair D, and McManus DP. 1995. A molecular phylogeny of the human schistosomes. *Molecular Phylogenetics and Evolution* 4:103-109.

Breugelmans K, Quintana Cardona J, Artois T, Jordaens K, and Backeljau T. 2012. First report of the exotic blue land planarian, *Caenoplana coerulea* (Platyhelminthes, Geoplanidae), on Menorca (Balearic Islands, Spain). *Zookeys* 199:91-105.

Carbayo F, Alvarez-Presas M, Jones HD, and Riutort M. 2016. The true identity of *Obama* (Platyhelminthes: Geoplanidae) flatworm spreading across Europe. *Zoological Journal of the Linnean Society* 177:5–28.

Chaisiri K, Dusitsittipon S, Panitvong N, Ketboonlue T, Nuamtanong S, Thaenkham U, Morand S, and Dekumyoy P. 2019. Distribution of the newly invasive New Guinea flatworm *Platydemus manokwari* (Platyhelminthes: Geoplanidae) in Thailand and its potential role as a paratenic host carrying *Angiostrongylus malaysiensis* larvae. *Journal of Helminthology* 93:711-719.

Clement M, Snell Q, Walke P, Posada D, and Crandall K. 2002. TCS: estimating gene genealogies. Proceedings of the 16th International Parallel and Distributed Process Symposium 2:184. Fort Lauderdale, Florida.

Cuevas-Caballé C, Riutort M, and Álvarez-Presas M. 2019. Diet assessment of two land planarian species using high-throughput sequencing data. *Scientific Reports* 9:8679.

de Beauchamp P. 1962. *Platydemus manokwari* n. sp., planaire terrestre de la Nouvelle-Guinée Hollandaise. *Bulletin de la Societe Zoologique de France* 87:609-615.

Dendy A. 1892. Short descriptions of new Land Planarians. *Proceedings of the Royal Society of Victoria* 4:35-38.

Dendy A. 1895. Notes on New Zealand Land Planarians: Part II. *Transactions of the Royal Society of New Zealand* 28:210-214.

European Union. 2019. Commission implementing regulation (EU) 2019/1262 of 25 July 2019 amending Implementing Regulation (EU) 2016/1141 to update the list of invasive alien species of Union concern. *Official Journal of the European Union*:26.27.2019, 2199/2011.

Gabbatiss J. 2018. Giant predatory worms invading France and threatening local wildlife. The Independent. London, UK.

Guarino B. 2018. Giant predatory worms invaded France, but scientists just noticed them. Washington Post. Washington, DC.

Hasegawa M, Kishino H, and Yano T-a. 1985. Dating of the human-ape splitting by a molecular clock of mitochondrial DNA. *Journal of Molecular Evolution* 22:160-174.

Hu J, Yang M, Ye ER, Ye Y, and Niu Y. 2019. First record of the New Guinea flatworm *Platydemus manokwari* (Platyhelminthes, Geoplanidae) as an alien species in Hong Kong Island, China. *Zookeys* 873:1-7.

Jones HD. 1999. A new genus and species of terrestrial planarian (Platyhelminthes; Tricladida; Terricola) from Scotland, and an emendation of the genus *Artioposthia*. *Journal of Natural History* 33:387-394.

Jones HD. 2019. Another alien terrestrial planarian in the United Kingdom: *Australopacifica atrata* (Steel, 1897)(Platyhelminthes: Tricladida: Continenticola). *Zootaxa* 4604:575-587.

Jones HD, Santoro G, Boag B, and Neilson R. 2001. The diversity of earthworms in 200 Scottish fields and the possible effect of New Zealand land flatworms (*Arthurdendyus triangulatus*) on earthworm populations. *Annals of Applied Biology* 139:75-92.

Jones HD, and Sluys R. 2016. A new terrestrial planarian species of the genus *Marionfyfea* (Platyhelminthes: Tricladida) found in Europe. *Journal of Natural History* 50:2673-2690.

Justine J-L. 2018. Des vers géants prédateurs envahissent les jardins français. Dans l'indifférence. . *The Conversation, 22 mai 2018* https://theconversationcom/des-vers-geants-predateurs-envahissent-les-jardins-francais-dans-lindifference-96241.

Justine J-L. 2019a. Analytics for paper published in The Conversation "Des vers géants prédateurs ..." May 2018. Dataset. https://doi.org/10.6084/m9.figshare.10271432.v1.

Justine J-L. 2019b. Plathelminthes terrestres invasifs. Blog (in French). https://sites.google.com/site/jljjustine/plathelminthe-terrestre-invasif.

Justine J-L, Lemarcis T, Gerlach J, and Winsor L. 2018a. First report of the land planarian *Endeavouria septemlineata* (Hyman, 1939) (Platyhelminthes, Tricladida, Continenticola, Geoplanidae) in French Polynesia. *Zootaxa* 4450:297-300.

Justine J-L, Théry T, Gey D, and Winsor L. 2019. First record of the invasive land flatworm *Bipalium adventitium* (Platyhelminthes, Geoplanidae) in Canada. *Zootaxa* 4656:591-595.

Justine J-L, Thévenot J, and Winsor L. 2014a. Les sept plathelminthes invasifs introduits en France. *Phytoma*:28-32 doi:10.6084/m6089.figshare.1447202.

Justine J-L, Winsor L, Barrière P, Fanai C, Gey D, Han AWK, La Quay-Velazquez G, Lee BPY-H, Lefevre J-M, Meyer J-Y, Philippart D, Robinson DG, Thévenot J, and Tsatsia F. 2015. The invasive land planarian *Platydemus manokwari* (Platyhelminthes, Geoplanidae): records from six new localities, including the first in the USA. *PeerJ* 3:e1037.

Justine J-L, Winsor L, Gey D, Gros P, and Thévenot J. 2014b. The invasive New Guinea flatworm *Platydemus manokwari* in France, the first record for Europe: time for action is now. *PeerJ* 2:e297.

Justine J-L, Winsor L, Gey D, Gros P, and Thévenot J. 2018b. Giant worms *chez moi*! Hammerhead flatworms (Platyhelminthes, Geoplanidae, *Bipalium* spp., *Diversibipalium* spp.) in metropolitan France and overseas French territories. *PeerJ* 6:e4672.

Kimura M. 1980. A simple method for estimating evolutionary rates of base substitutions through comparative studies of nucleotide sequences. *Journal of Molecular Evolution* 16:111-120.

Kosmala M, Wiggins A, Swanson A, and Simmons B. 2016. Assessing data quality in citizen science. *Frontiers in Ecology and the Environment* 14:551-560.

Kubota S, and Kawakatsu M. 2010. Distribution record of a single species of the collective group *Diversibipalium* (Plathelminthes, Tricladida, Continenticola, Geoplanidae, Bipaliinae) in Wakayama Prefecture, Honshu, Japan, with a taxonomic note of new higher classification of the Tricladida. *Nanki Seibutsu* 52:97-101.

Kullenberg C, and Kasperowski D. 2016. What is citizen science? – a scientometric meta-analysis. *PLoS ONE* 11:e0147152.

Kumar S, Stecher G, and Tamura K. 2016. MEGA7: Molecular Evolutionary Genetics Analysis version 7.0 for bigger datasets. *Molecular Biology and Evolution* 33:1870-1874.

Lago-Barcia D, Fernández-Álvarez FÁ, Brusa F, Rojo I, Damborenea C, Negrete L, Grande C, and Noreña C. 2019. Reconstructing routes of invasion of *Obama nungara* (Platyhelminthes: Tricladida) in the Iberian Peninsula. *Biological Invasions* 21:289–302.

Lago-Barcia D, Fernández-Álvarez FA, Negrete L, Brusa F, Damborenea C, Grande C, and Noreña C. 2015. Morphology and DNA barcodes reveal the presence of the non-native land planarian *Obama marmorata* (Platyhelminthes: Geoplanidae) in Europe. *Invertebrate Systematics* 29:12-22.

Lázaro EM, Sluys R, Pala M, Stocchino GA, Baguñà J, and Riutort M. 2009. Molecular barcoding and phylogeography of sexual and asexual freshwater planarians of the genus *Dugesia* in the Western Mediterranean (Platyhelminthes, Tricladida, Dugesiidae). *Molecular Phylogenetics and Evolution* 52:835-845.

Leigh JW, and Bryant D. 2015. POPART: full-feature software for haplotype network construction. *Methods in Ecology and Evolution* 6:1110-1116.

Littlewood DTJ, Rohde K, and Clough KA. 1997. Parasite speciation within or between host species? - Phylogenetic evidence from site-specific polystome monogeneans. *International Journal for Parasitology* 27:1289-1297.

Makino N, and Shirasawa Y. 1983. Morphological and ecological comparison with two new species of elongated slender land planarians have several stripes and their new scientific names. *Bulletin of Tokyo Medical College* 9:69-83 [In Japanese, English summary].

Mateos E, Tudó A, Álvarez-Presas M, and Riutort M. 2013. Planàries terrestres exòtiques a la Garrotxa. *Annals de la Delegació de la Garrotxa de la Institució Catalana d'Història Natural* 6:67-73.

Mazza G, Menchetti M, Sluys R, Solà E, Riutort M, Tricarico E, Justine J-L, Cavigioli L, and Mori E. 2016. First report of the land planarian *Diversibipalium multilineatum* (Makino & Shirasawa, 1983) (Platyhelminthes, Tricladida, Continenticola) in Europe. *Zootaxa* 4067:577–580.

Morin H. 2018. Des vers géants envahissent la France. Le Monde. Paris, France.

Moseley H. 1877. Notes on the structure of several forms of land planarians, with a description of two new genera and several new species, and a list of all species at present known. *Quarterly Journal of Microscospical Science* 17:273-292.

Moseley HN. 1878. Description of a new species of land-planarian from the hothouses at Kew Gardens. *Annals and Magazine of Natural History* 1:237-239.

Prozorova LA, and Ternovenko VA. 2018. [Rare and new species from the Far Eastern Marine Reserve. 2. Land Planarians (Platyhelminthes: Tricladida: Continenticola)] Редкие и новые виды организмов Дальневосточного морского заповедника. 2. Наземные планарии (Platyhelminthes: Tricladida: Continenticola). *Biota and Environment* 3:54-59.

Rodríguez-Cabrera TM, and Torres J. 2019. New locality records of *Bipalium kewense* (Platyhelminthes: Tricladida: Geoplanidae) in Cuba. *Poeyana* 508:38-41.

Saitou N, and Nei M. 1987. The neighbor-joining method: a new method for reconstructing phylogenetic trees. *Molecular Biology and Evolution* 4:406-425.

Sluys R. 2016. Invasion of the Flatworms. *American Scientist* 104:288-295.

Sluys R. 2019. Klasse Turbellaria – Strudelwürmer. In: Klausnitzer B, ed. *Stresemann - Exkursionsfauna von Deutschland Band 1: Wirbellose (ohne Insekten)*. Berlin, Heidelberg: Springer Berlin Heidelberg, 59-69.

Soors J, Van den Neucker T, Halfmaerten D, Neyrinck S, and De Baere M. 2019. On the presence of the invasive planarian *Obama nungara* (Carbayo, Álvarez-Presas, Jones & Riutort, 2016) (Platyhelminthes: Geoplanidae) in an urban area in Belgium. *Belgian Journal of Zoology* 149:43-47.

Steel T. 1897. Australian land planarians: descriptions of new species and notes on collecting and preserving. *Proceedings of the Linnean Society of New South Wales* 22:104–119.

Tamura K. 1992. Estimation of the number of nucleotide substitutions when there are strong transition-transversion and G+C-content biases. *Molecular Biology and Evolution* 9:678-687.

Tamura K, Nei M, and Kumar S. 2004. Prospects for inferring very large phylogenies by using the neighbor-joining method. *Proceedings of the National Academy of Sciences of the United States of America* 101:11030-11035.

Vardinoyannis K, and Alexandrakis G. 2019. First record of the land planarian *Caenoplana bicolor* (Graff, 1899) (Platyhelminthes, Tricladida, Continenticola) in Greece. *BioInvasions Records* 8:500-504.

Vidard M. 2018. La menace des vers géants. France Inter (National French Radio). Paris: Radio France.

von Graff L. 1899. *Monographie der Turbellarien. II. Tricladida, Terricola (Landplanarien)*. Leipzig: Englemann.

Winsor L. 1991. A provisional classification of Australian terrestrial geoplanid flatworms (Tricladida: Terricola: Geoplanidae). *Victorian Naturalist (Blackburn)* 108:42-49.
