# Supplementary figures and images for: Obama chez moi! The invasion of metropolitan France by the land planarian Obama nungara (Platyhelminthes, Geoplanidae)
Source: PeerJ. 2020 Feb 6;8:e8385. doi: 10.7717/peerj.8385 (PMC7007977; doi:10.7717/peerj.8385)

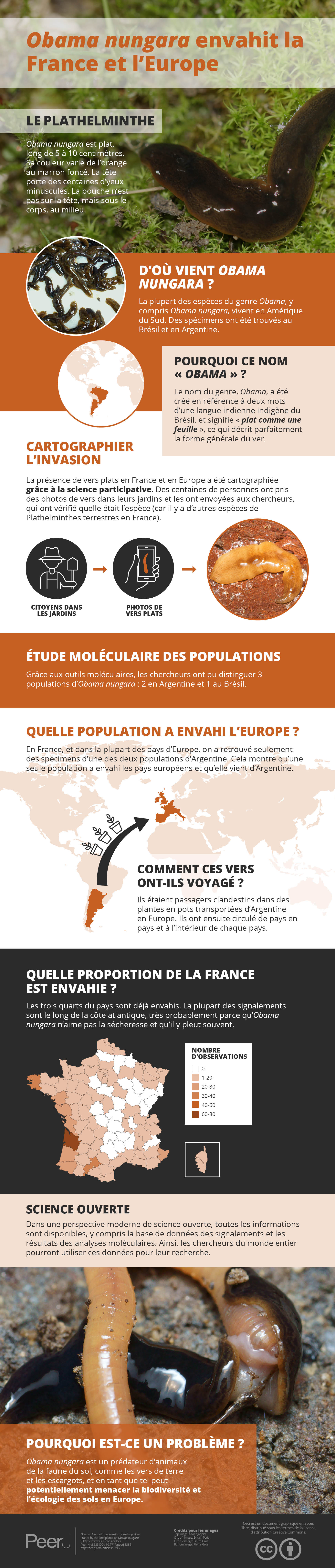

Supplement: Supplemental Information 5 [file peerj-08-8385-s005.jpg]
