# Supplementary material for: Obama chez moi! The invasion of metropolitan France by the land planarian Obama nungara (Platyhelminthes, Geoplanidae)
Source: PeerJ. 2020 Feb 6;8:e8385. doi: 10.7717/peerj.8385 (PMC7007977; doi:10.7717/peerj.8385)
Supplement: Supplemental Information 6 [file peerj-08-8385-s006.pdf]

Obama nungara envahit la France et l'Europe

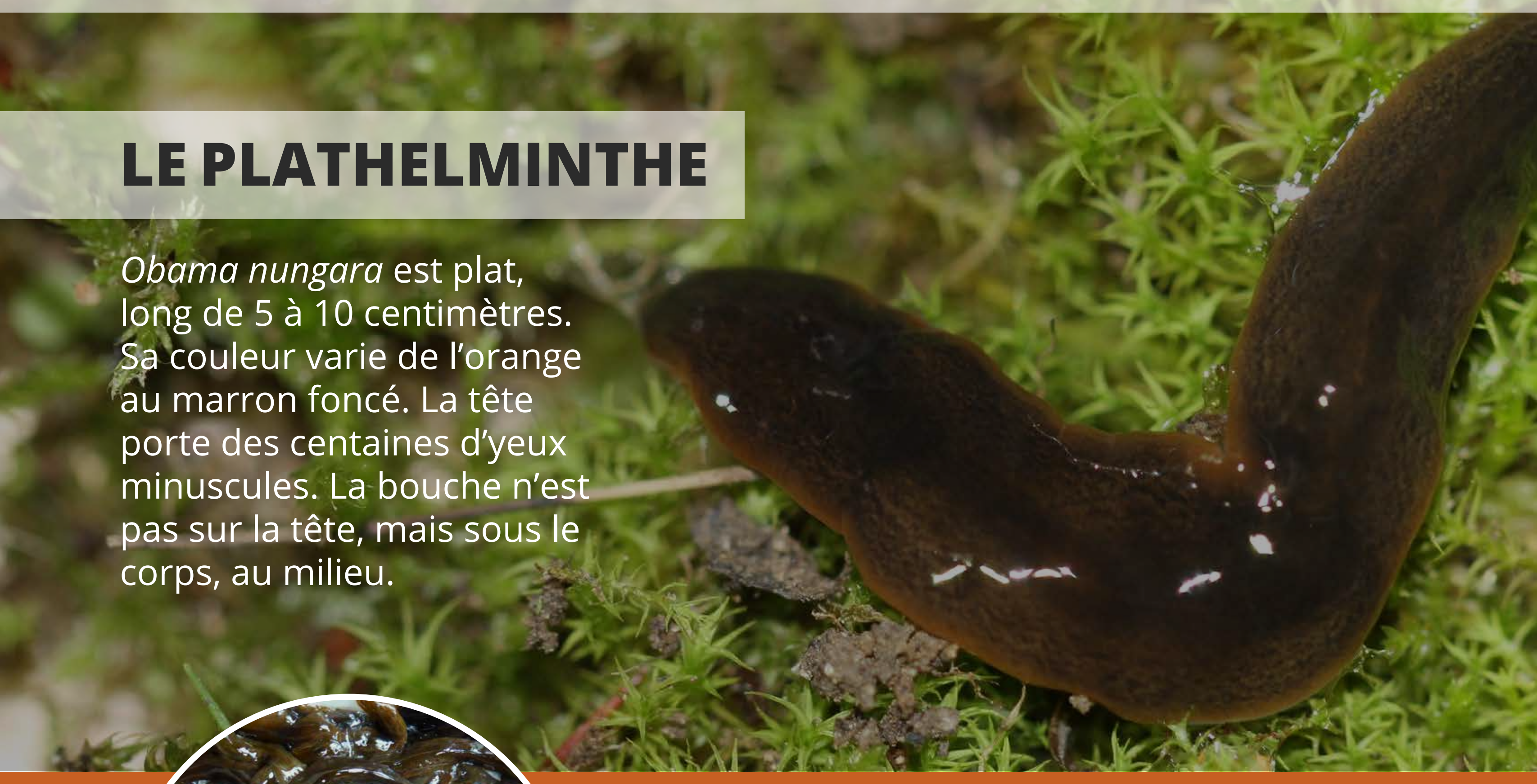

LE PLATHELMINTHE

Obama nungara est plat, long de 5 à 10 centimètres. Sa couleur varie de l'orange au marron foncé. La tête porte des centaines d'yeux minuscules. La bouche n'est pas sur la tête, mais sous le corps, au milieu.

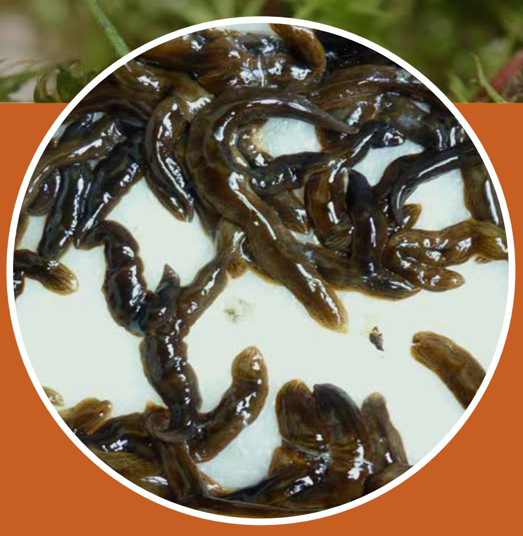

D'OÙ VIENT *OBAMA NUNGARA* ?

La plupart des espèces du genre *Obama*, y compris *Obama nungara*, vivent en Amérique du Sud. Des spécimens ont été trouvés au Brésil et en Argentine.

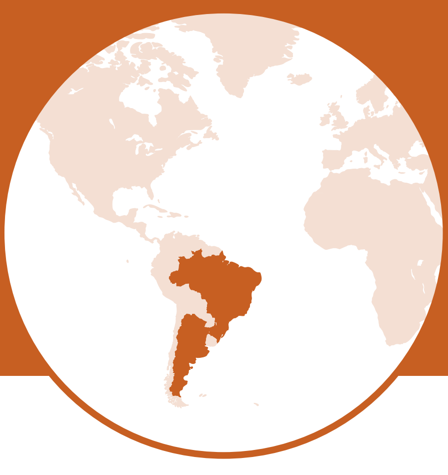

POURQUOI CE NOM « *OBAMA* » ?

Le nom du genre, *Obama*, a été créé en référence à deux mots d'une langue indienne indigène du Brésil, et signifie « ***plat comme une feuille*** », ce qui décrit parfaitement la forme générale du ver.

CARTOGRAPHIER L'INVASION

La présence de vers plats en France et en Europe a été cartographiée **grâce à la science participative**. Des centaines de personnes ont pris des photos de vers dans leurs jardins et les ont envoyées aux chercheurs, qui ont vérifié quelle était l'espèce (car il y a d'autres espèces de Plathelminthes terrestres en France).

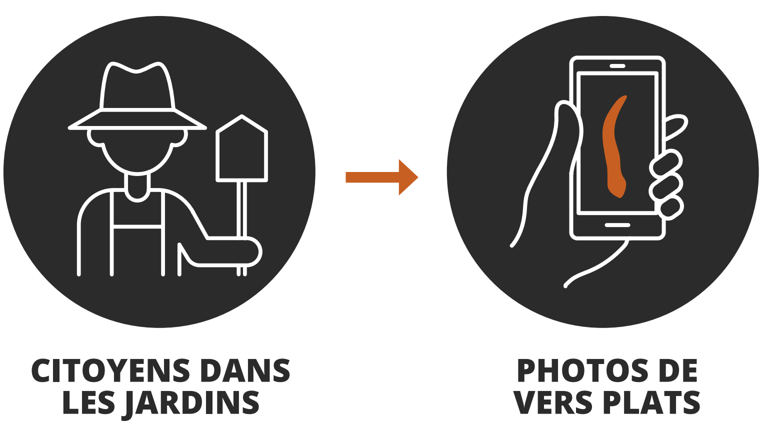

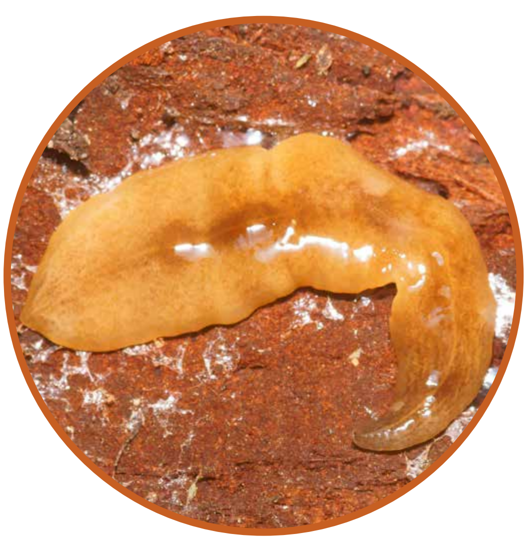

ÉTUDE MOLÉCULAIRE DES POPULATIONS

Grâce aux outils moléculaires, les chercheurs ont pu distinguer 3 populations d'*Obama nungara* : 2 en Argentine et 1 au Brésil.

QUELLE POPULATION A ENVAHI L'EUROPE ?

En France, et dans la plupart des pays d'Europe, on a retrouvé seulement des spécimens d'une des deux populations d'Argentine. Cela montre qu'une seule population a envahi les pays européens et qu'elle vient d'Argentine.

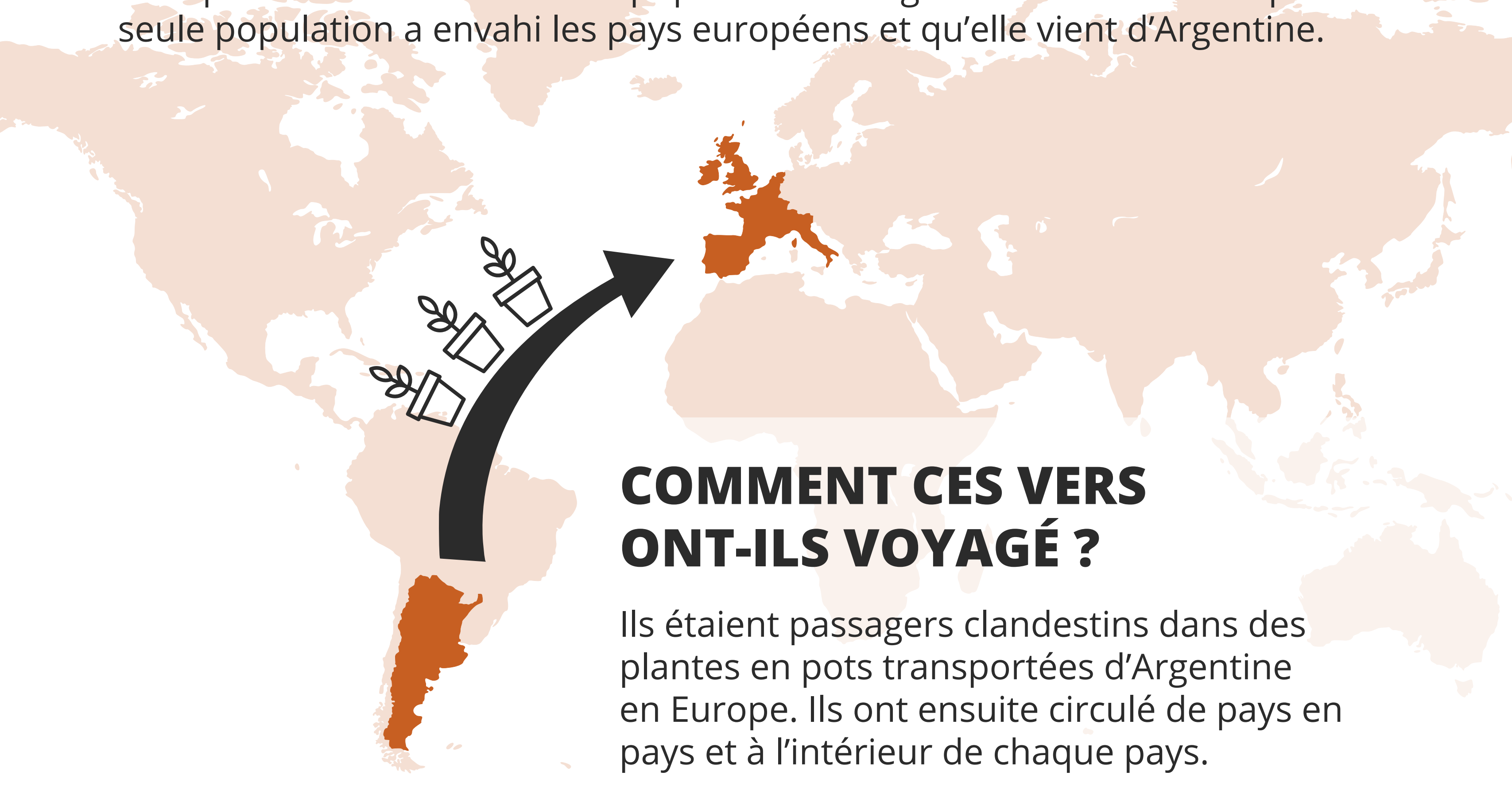

COMMENT CES VERS ONT-ILS VOYAGÉ ?

Ils étaient passagers clandestins dans des plantes en pots transportées d'Argentine en Europe. Ils ont ensuite circulé de pays en pays et à l'intérieur de chaque pays.

QUELLE PROPORTION DE LA FRANCE EST ENVAHIE ?

Les trois quarts du pays sont déjà envahis. La plupart des signalements sont le long de la côte atlantique, très probablement parce qu'*Obama nungara* n'aime pas la sécheresse et qu'il pleut souvent.

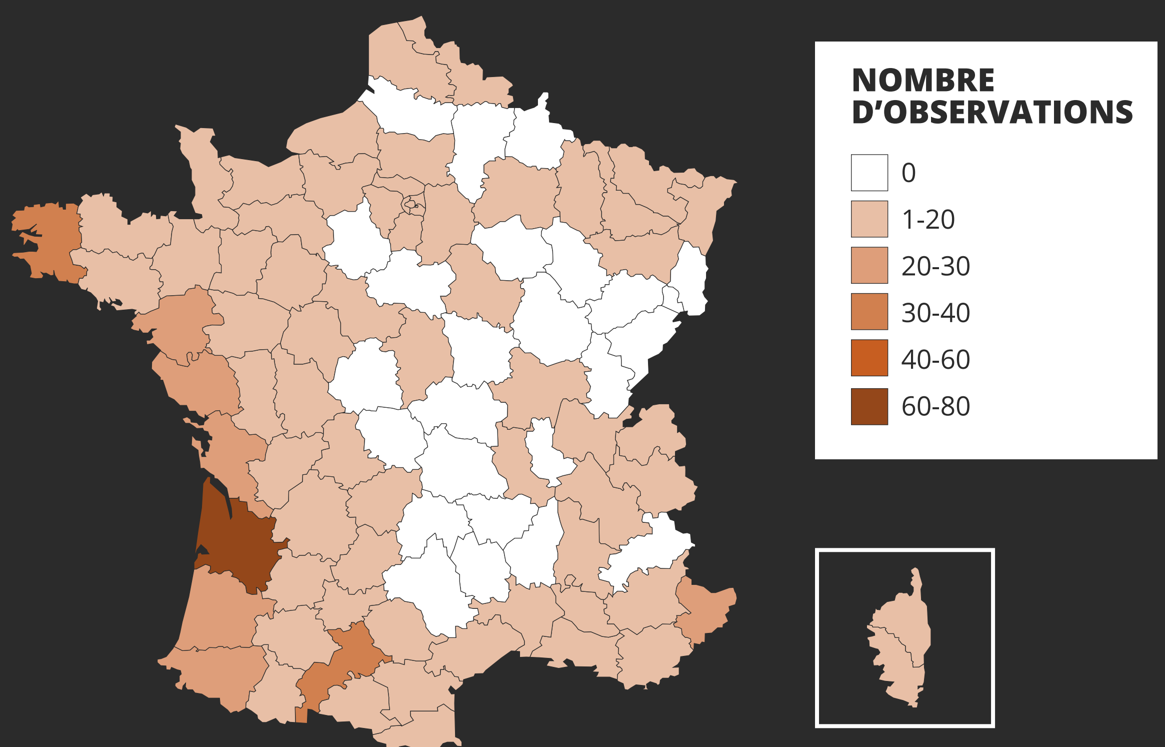

SCIENCE OUVERTE

Dans une perspective moderne de science ouverte, toutes les informations sont disponibles, y compris la base de données des signalements et les résultats des analyses moléculaires. Ainsi, les chercheurs du monde entier pourront utiliser ces données pour leur recherche.

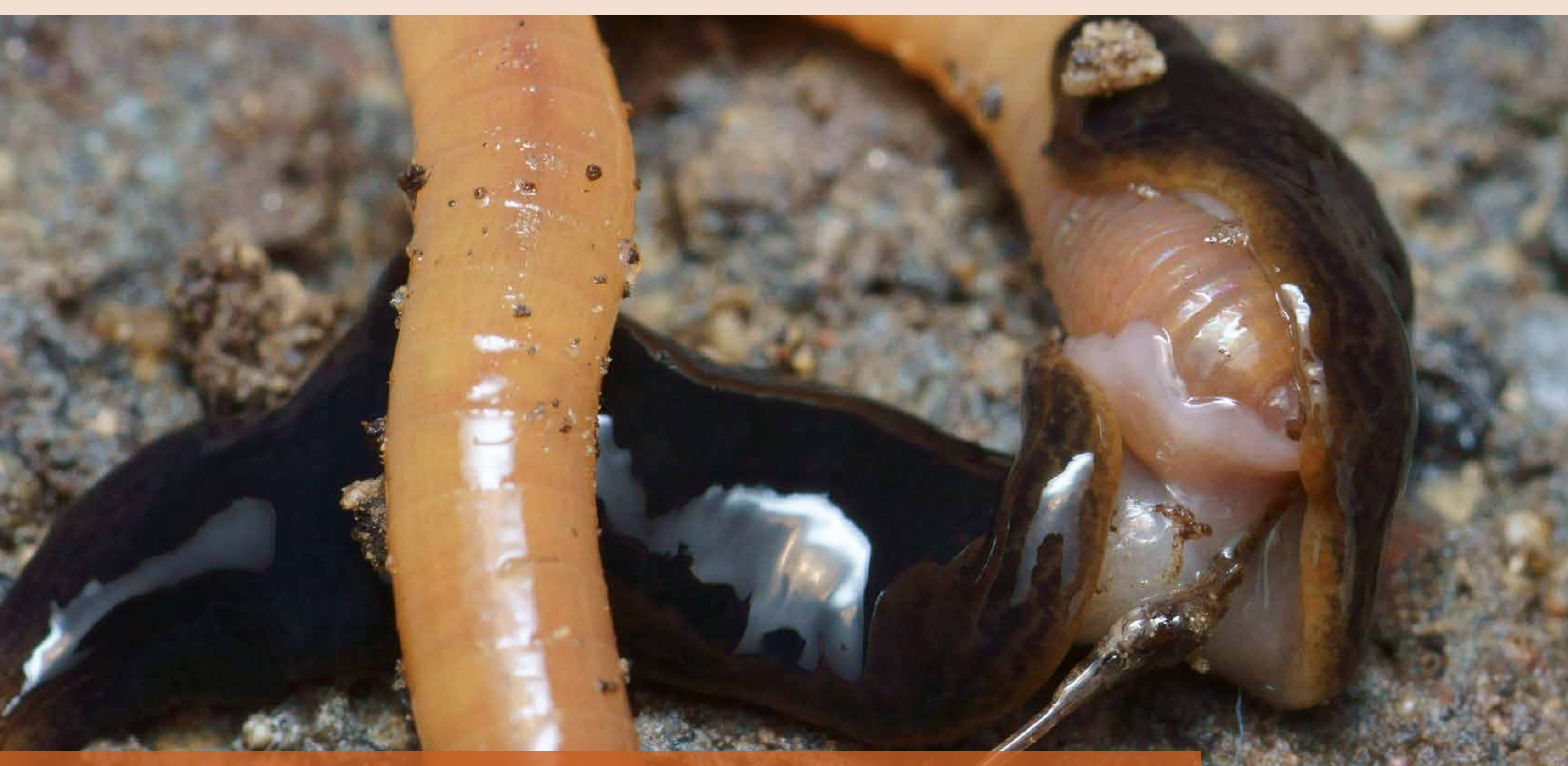

POURQUOI EST-CE UN PROBLÈME ?

Obama nungara est un prédateur d'animaux de la faune du sol, comme les vers de terre et les escargots, et en tant que tel peut **potentiellement menacer la biodiversité et l'écologie des sols en Europe.**

PeerJ
Obama chez moi! The invasion of metropolitan France by the land planarian *Obama nungara* (Platyhelminthes, Geoplanidae)
PeerJ 4:e8385 DOI: 10.7717/peerj.8385
http://peerj.com/articles/8385/

Crédits pour les images
Top image: Xavier Jappiot
Circle 1 image: Sylvain Petiet
Circle 2 image: Pierre Gros
Bottom image: Pierre Gros

Ceci est un document graphique en accès libre, distribué sous les termes de la licence d'attribution Creative Commons.
